# Inhibition Kinetics and Theoretical Studies on *Zanthoxylum chalybeum* Engl. Dual Inhibitors of α-Glucosidase and α-Amylase

**DOI:** 10.3390/jox13010009

**Published:** 2023-02-21

**Authors:** Njogu M. Kimani, Charles O. Ochieng, Mike Don Ogutu, Kevin Otieno Yamo, Joab Otieno Onyango, Cleydson B. R. Santos

**Affiliations:** 1 Department of Physical Sciences, University of Embu, Embu P.O. Box 6-60100, Kenya; 2 Department of Chemistry, Maseno University, Maseno P.O. Box 333-40105, Kenya; 3 School of Chemical Sciences and Technology, Technical University of Kenya, Nairobi P.O. Box 52428-00200, Kenya; 4 Graduate Program in Biotechnology and Biodiversity-Network BIONORTE, Federal University of Amapá, Macapá 68903-419, AP, Brazil; 5 Laboratory of Modeling and Computational Chemistry, Department of Biological and Health Sciences, Federal University of Amapá, Macapá 68902-280, AP, Brazil

**Keywords:** diabetes, α-glucosidase, α-amylase, molecular docking, *Zanthoxylum chalybeum*, ADMET profiling

## Abstract

Compounds from *Zanthoxylum chalybeum* Engl. were previously reported for inhibitory activities of amylase and glucosidase enzymatic action on starch as a preliminary study toward the establishment of a management strategy against postprandial hyperglycemia, however, the inhibitory kinetics and molecular interaction of these compounds were never established. A study was thus designed to establish the inhibitory kinetics and in silico molecular interaction of α-glucosidase and α-amylase with *Z. chalybeum* metabolites based on Lineweaver–Burk/Dixon plot analyses and using Molecular Operating Environment (MOE) software, respectively. Skimmianine (**5**), Norchelerythrine (**6**), 6-Acetonyldihydrochelerythrine (**7**), and 6-Hydroxy-N-methyldecarine (**8**) alkaloids showed mixed inhibition against both α-glucosidase and α-amylase with comparable *K_i_* to the reference acarbose (*p* > 0.05) on amylase but significantly higher activity than acarbose on α-glucosidase. One phenolic 2,3-Epoxy-6,7-methylenedioxyconiferol (**10**) showed a competitive mode of inhibition both on amylase and glucosidase which were comparable (*p* > 0.05) to the activity of acarbose. The other compounds analyzed and displayed varied modes of inhibition between noncompetitive and uncompetitive with moderate inhibition constants included chaylbemide A (**1**), chalybeate B (**2**) and chalybemide C (**3**), fagaramide (**4**), ailanthoidol (**9**), and sesame (**11**). The important residues of the proteins α-glucosidase and α-amylase were found to have exceptional binding affinities and significant interactions through molecular docking studies. The binding affinities were observed in the range of −9.4 to −13.8 and −8.0 to −12.6 relative to the acarbose affinities at −17.6 and −20.5 kcal/mol on α-amylase and α-glucosidase residue, respectively. H-bonding, π-H, and ionic interactions were noted on variable amino acid residues on both enzymes. The study thus provides the basic information validating the application of extracts of *Z. chalybeum* in the management of postprandial hyperglycemia. Additionally, the molecular binding mechanism discovered in this study could be useful for optimizing and designing new molecular analogs as pharmacological agents against diabetes.

## 1. Introduction

Diabetes mellitus (DM), a metabolic condition displayed by extraordinary amounts of plasma glucose (hyperglycemia), is closely linked to death and morbidity worldwide. It is a condition brought on by insufficient or excessive insulin secretion, insulin resistance, or both [1,2]. Predisposing factors of DM can be traced to environmental and genetic factors, namely, changes in physical activity and dietary habits, age, resistance to insulin, and diabetes in the family medical history [3].

In spite of the failure to discover an all-around therapeutic remedy, a number of management options have been discovered alongside insulin which have enhanced the management of DM [4]. Different categories of antidiabetic drugs are available which exert their action by boosting the body’s insulin production, improving the body’s sensitivity to insulin or reducing insulin resistance, and reducing intestinal glucose absorption [5]. The later therapeutic route may entail modulation of the enzyme α-amylase and α-glucosidase to delay the glucose absorption rate so as to maintain an optimal blood glucose level in DM patients. However, the major drawback of these drugs is their non-specificity in targeting different glucosidases. Practical examples include miglitol and acarbose. They are effective at decreasing glucose absorption through the inhibition of the activity of the α-glucosidases found in the small intestinal brush barrier, although they often cause diarrhea, flatulence, and abdominal bloating [6]. Metformin has been demonstrated as a better medication for DM, although is not recommended for patients with decreased renal or hepatic function [7].

**Figure 1:**
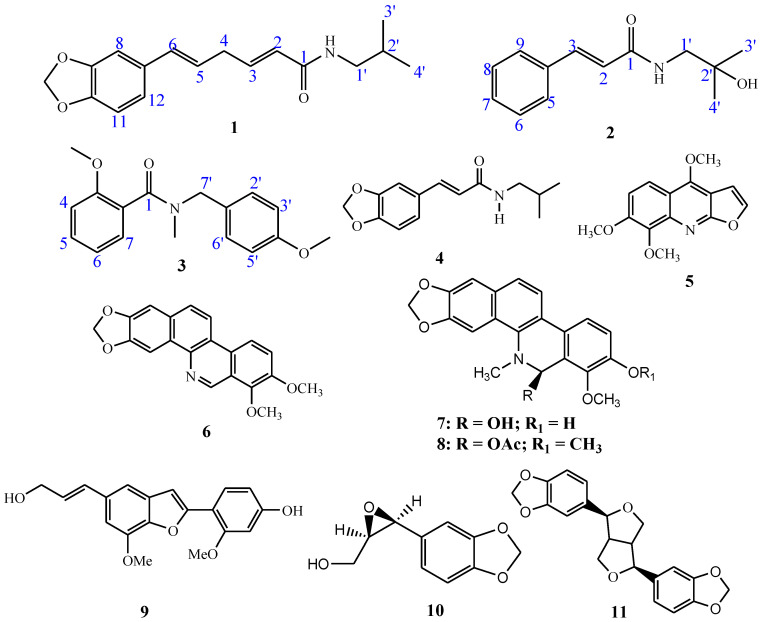
Identified compounds from *Z. chalybeum*’s root bark.

The aforementioned negative side effects of these medications have prompted researchers to look for alternate treatments with fewer severe drawbacks, especially those derived from natural drug reservoirs such as the metabolites of medicinal plants. In order to alleviate ailments and ease human suffering, herbal medicines and natural products have been employed as a source of medicine for a long time. As a result, interest in phytomedicine is rising; plant extracts have the potential to be safer, are more readily available, are less expensive, and have fewer negative side effects than synthetic antihyperglycemic medications [8]. However, the scope of discovering novel natural compounds with pharmacological importance in order to control type II diabetic mellitus (T2DM) is still constrained owing to the lack of a sufficient mechanism-based comprehensive investigation of these phytopharmaceuticals. Following the application of the root bark and stem of *Z. chalybeum* Engl. (Rutaceae) by traditional healers, a thorough bioassay investigation showed the extracts have an antihyperglycemic potential against streptozotocin- and alloxan-induced diabetic rats [9,10,11]. Subsequently, bioactivity-guided isolation resulted in the identification of some of the bioactive compounds including chaylbemide A (**1**), chalybemide B (**2**) and chalybemide C (**3**), fagaramide (**4**); skimmianine (**5**), norchelerythrine (**6**), 6-acetonyldihydrochelerythrine (**7**) and 6-hydroxy-N-methyldecarine (**8**), ailanthoidol (**9**), 2,3-epoxy-6,7-methylenedioxyconiferol (**10**) and sesamine (**11**) with the structures shown in Figure 1. These compounds have been shown to inhibit the enzymes α-glycosidase and α-amylase with IC_50_ values between 43.22 and 49.36 μM at comparable levels to (*p* > 0.05) the positive control acarbose which has IC_50_ values of 42.67 and 44.88 μM against α-amylase and α-glycosidase, respectively [12]. Such results established the ability of *Z. chalybuem* against DM; however, the study failed to establish the possible mechanism of interaction between the enzymes and the inhibitors (compounds), thus necessitating further investigation on the mode of actions via inhibition kinetics and molecular interaction studies.

The lock-and-key mechanism, where the lock encrypts the protein and the ligand is the key, is analogous to protein–ligand interaction. Hydrophobic contact appears to be the main mechanism promoting binding. By using chemoinformatic/bioinformatics tools, in silico techniques assist in finding pharmacological targets. Additionally, they can be used to discover potential active sites in target structures, create candidate compounds, dock the target with these ligands, use resulting binding affinities to order the ligands, and to enhance binding capabilities, further modify the molecules [13]. In an effort to create novel anti-diabetic drugs, in silico molecular modeling and analysis has been used to establish the potential mode of interaction of therapeutic agents with molecular receptors [14] to confirm the classical experimental bioassays. In that respect, a study to establish the mechanism of action based on inhibition constants and the in silico molecular interaction analysis of these *Z. chalybeum* metabolites against α-glucosidase and α-amylase was completed and results are reported herewith.

## 2. Materials and Methods

### 2.1. Isolation of Study Compounds and Their Kinetic Analyses

The compounds under study were isolated from the root barks of *Z. chalybeum.* Briefly, the root barks were chopped into small pieces separately, air-dried at room temperature under shade for 21 days, and ground into a fine powder using an electric pulverizer. The powdered root bark (0.8 kg) was exhaustively extracted with 95% aqueous methanol (4 × 1.5 L) and filtered to afford a 30 g crude sample. The crude sample was partitioned into total alkaloid extraction and nonalkaloid fraction, followed by a series of chromatographic procedures that led to the isolation of eleven pure compounds as described by Ochieng et al., 2020 [12]. Furthermore, the structures of the eleven compounds were elucidated following spectroscopic techniques as described by Ocheing et al., 2020 [12].

Mode of compound inhibition against porcine pancreas α-amylase and yeast α-glucosidase were determined at increasing substrate, pNPG, concentrations (0.25, 0.5, 1, 2, and 5 mM), both when the pure compounds **1**–**11** and acarbose were present at 0, 0.5, 1, 2.5, 10, and 20 mM and in their absence. Using Lineweaver–Burk plots, the mode of inhibition was established, followed by secondary plots (Dixon plots) depending on the established mode of inhibition. The following equation was used to obtain the inhibition constants (*K_i_*) [15]:

$$v=V_{max}\;\times \frac{S}{K_{m}\left(1+\frac{\left[I\right]}{K_{i}}\right)}+S\left(1+\frac{\left[I\right]}{\alpha K_{i}}\right)$$

where *S* and *I* are the concentrations of the substrate and inhibitor, respectively; *V_max_* is the maximum velocity; *K_m_* is the Michaelis–Menten constant; *K_i_* is the competitive inhibition constant; and *αK_i_* is the uncompetitive inhibition constant.

### 2.2. Statistical Analysis

A computer application for nonlinear regressions on the MS-Excel-2019 version was used to evaluate the kinetic data. Lineweaver–Burk plots on monoreplicate tests were performed followed by Dixon secondary plots to determine the inhibition constants. The means of the observed triplicate inhibition constants were subjected to analysis of variance with Tukey HSD/Tukey Kramer post-analysis to compare means. The least significant difference was considered at *p* < 0.05 and the coefficient of determination (R^2^) was obtained as the average of the regression curves from Dixon plots of individual experiments.

### 2.3. In Silico Method

The Molecular Operating Environment (MOE) software v. 2015.10 from the Chemical Computing Group, Montreal, QC, Canada and the incorporated Merck Molecular Force Field (MMFF94x) were used for all in silico studies [16].

#### 2.3.1. Ligands Preparation

Compounds **1**–**11** were obtained from the literature, and their 2D molecular graphs were sketched in ChemDraw Ultra Ver. 12.0 and saved as MDL files (.sdf). The .sdf file format of acarbose, the reference molecule, was retrieved from NCBI PubChem [17]. All the molecules were then imported into MOE where three-dimensional (3D) molecular models of each were generated. The MMFF94x force field was then used to optimize the generated geometries and subsequently subjected to a low-mode molecular dynamics conformational search (LowModeMD) to obtain the most favorable conformers for each ligand. An energy threshold of 5 kcal/mol above the lowest energy conformation was applied and the conformation limit was set to 10 for each ligand. The resulting conformers with the lowest force field energy were minimized using the AM1 Hamiltonian (MOPAC module of MOE), and the minimized geometries then saved into a MOE database for further action.

#### 2.3.2. Drug-Likeness Predictions and Structural Skeleton Similarity Analysis

The 11 ligands and acarbose were evaluated using Lipinski’s rule of five, taking the following factors into consideration: lipophilicity, molecular weight, and the amount of hydrogen bond acceptors and donors [18]. To establish the structural skeleton similarity common to these molecules, further analysis of their physicochemical parameters was completed. This was executed in DataWarrior software [19], a flexible, interactive, and chemistry-aware tool for the viewing and interpretation of chemical data. The existence of eight structural properties—electronegative atoms, carbo rings, aromatic carbon atoms, H-donor atoms, H-acceptor atoms, heterorings, rotatable bonds, and ring closures—was enumerated and examined after the compounds were subjected to DataWarrior for structure analysis.

#### 2.3.3. ADME/Tox Prediction

The compounds’ absorption, distribution, metabolism, and excretion (as well as toxicological; ADME/Tox) characteristics were predicted by means of the PreADMET online server [20] and SwissADME server [21]. This server computes pharmacokinetic properties such as Human Intestinal Absorption (HIA), the permeability of Caco-2 cells in vitro (PCaco-2), skin permeability (PSkin), plasma protein-binding (PPB), and permeation through the blood–brain barrier (CBrain/CBlood). The toxicity was predicted using PreADMET and pro-Tox II servers [22].

#### 2.3.4. 3D Protein Structures Preparation

To predict, in silico, the potential interactions of the ligands with the enzymes α-glucosidase and α-amylase (PDB-IDS: 2QMJ and 7TAA, respectively) [23,24], 3D structures of the receptors were obtained from the Research Collaboratory for Structural Bioinformatics (RCSB) Protein Data Bank (PDB) [25]. The protein models were first prepared by removing all water molecules (MOE: SEQ) and correcting all incomplete and omitted residual amino acids that could result from the X-ray crystallographic data identified through the MOE software (Compute, Prepare, Structure Preparation, Correct). At 27 °C and pH 7.0, the models were virtually titrated to modify the ionization step of acidic and basic side chains of the amino acid. The models were then protonated accordingly in MOE software. Following that, the energy of the enzyme models was decreased in order to minimize the structure of the protein within the confines of the allocated force field. The reduction was completed slowly by setting all heavy atom sites and only permitting a steady increase within a radius of 0.5 to 1.5 Å in MOE software following the steps Compute, Energy Minimize, and Tether Atoms. This was to ensure no major variation in the protein structure as determined by the experiment. Finally, without any constraints, an energy minimization was performed, resulting in totally relaxed protein structures for further investigation.

#### 2.3.5. Docking Simulation

Each molecule was simulated to determine its optimal orientation within each protein model binding site (MOE: Compute, Dock). Before docking, α-glucosidase and α-amylase amino-acid residues’ binding interactions with acarbose were investigated. The protein sites for each enzyme model consisted of Asp203, Asp542, Asp327, His600, and Arg526 residues for α-glucosidase and Trp83, Asp340, Arg344, Arg204, Glu230, and Lys209 for α-amylase were selected and occupied by dummy atoms. To explore the binding modes of the 11 compounds with α-glucosidase and α-amylase proteins, 10 conformations of each ligand were docked into the respective enzyme’s chosen binding pocket using MOE-Dock module with conditions, placement: triangle matcher, rescoring: London dG, refinement: forcefield, retain: 10. RMSD (root mean square deviation) values, and docking scores of ligands’ top-ranked conformers were utilized to investigate their binding mechanisms. The determined Gibbs energy (MOE: London DG) resulting from the produced complexes of the enzymes and ligands (docking poses) were applied as the scoring parameters in this regard. Each complex’s S-score relates to its virtual free energy in kcal/mol. As a spontaneous reaction is indicated by a larger negative result, all docking poses were arranged in increasing S-score order.

Before conducting molecular docking studies on the two enzymes, the docking protocol employed in this work was validated. To accomplish this, the co-crystalized ligand (acarbose) was re-docked into the binding sites of the enzymes, commonly referred to as self-dock. The docking simulation was carried out with the lowest energy conformer of the ligand docked into the active sites of the two proteins in various conformations, with the proteins treated as rigid entities (MOE: Rigid Receptor). The top ten hits from the docking simulation were used for further analysis.

**Figure 2:**
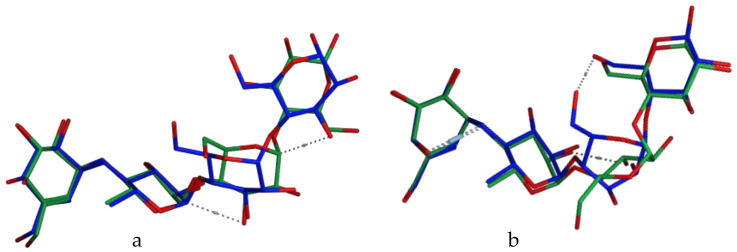
Superposed structures of acarbose after validation of the binding mode as obtained using the MOE software: in blue, the crystallographic pose; in green, the top-ranked docking pose. (**a**) α-glucosidase (PDB ID: 2QMJ); (**b**) α-Amylase (PDB ID: 7TAA).

The postures derived from the self-dock were compared with the experimental conformation (the crystallographic pose), yielding binding modes, and orientations that are as shown in Figure 2. Additionally, the re-docked data of the co-crystallized ligand–protein interactions strongly corresponded (see Table 1) with the original interactions from the crystal structure complexes; additionally, the co-crystallized ligands’ RMSD values 1.74 Å for α-amylase and 1.6 Å for α-glucosidase which are less than 2 Å, which is recommended [26,27,28,29,30,31], demonstrate that the docking approach was adequately validated. For each enzyme structure, the lowest S-score from each self-dock was chosen and used as the reference for all subsequent docking simulations with that protein.

## 3. Results and Discussion

### 3.1. Kinetic Analyses

Kinetic analysis based on both Lineweaver–Burk plots and Dixon plots revealed the modes of inhibition and the enzymes–inhibitor inhibition constants (Table 1) revealed that compounds showing mixed inhibitory modes such as Skimmianine (**5**), Norchelerythrine (**6**), 6-Acetonyldihydrochelerythrine (**7**), and 6-Hydroxy-N-methyldecarine (**8**) showed comparable *K_i_* to acarbose (*p* > 0.05) on amylase while the other six compounds which showed significantly (*p* < 0.05) low activity compared to acarbose displayed either noncompetitive or uncompetitive modes against α-amylase actions on starch. The same compounds showing mixed inhibition on amylase showed similar modes on α-glucosidase activities, with significantly (*p* < 0.05) higher *K_i_* values compared to *K_i_* of acarbose indicating a better inhibitory potential towards α-glucosidase than amylase. Conversely, the other compounds displaying non-competitive and uncompetitive inhibitions against α-glucosidase activities showed comparable *K_i_* values (*p* < 0.05) relative to acarbose. One compound 2,3-Epoxy-6,7-methylenedioxyconiferol (**10**) showed a competitive mode of inhibition both on α-amylase and α-glucosidase with *K_i_* of 5.54 ± 0.58 (R^2^ = 0.985) and 17.21 ± 0.15 (R^2^ = 0.8692), respectively, which were statistically comparable (*p* < 0.05) to that of acarbose thus indicative of the most active inhibitors from the *Z. chalybeum* extracts. Compound **10** and the alkaloids (**5, 6, 7,** and **8**) displaying competitive and mixed inhibitions, respectively, were thus invariably noted as the most potent inhibitors of both amylase and glucosidase. On the other hand, the remaining compounds **1**, **2**, **3**, **4**, **9**, and **11** showed varied modes of inhibition and associated dissociation constants towards the two enzymes which would be categorized as moderate inhibitory activities. Such preliminary kinetic results would thus be better confirmed with molecular interaction studies based on in silico experiments.

### 3.2. Drug-Likeness Predictions and Structural Skeleton Similarity Analysis

Lipinski’s rule of five and the Veber rules are closely linked to drug-likeness properties. Molecules with characteristics that match these rules could be deemed promising therapeutic candidates with high oral bioavailability. If a drug molecule fails to fulfill more than one of the five rules, it will have poor oral absorption. Lipinski’s rule of five states that a compound is orally bioactive when it has a molecular weight (MW) of 500 or less, a cLogP (partition coefficient between n-octanol and water) of 5 or less, a number of hydrogen bond donors (HBD) of no more than 5, a number of hydrogen bond acceptors (HBA) equal to or less than 10, and a number of rotatable bonds (RB) of 10 or less [32,33]. Unlike Lipinski’s rule of five, the Veber rules only specify two requirements for drug candidates to have excellent oral bioavailability. These requirements are that there are no more than ten rotatable bonds and that the polar surface area is at most 140 Å.

Drug likeness is a high degree of control of several molecular and structural characteristics that determine whether a given ligand is similar to approved drugs. These descriptors (molecule size, hydrogen bonding properties, hydrophobicity, flexibility, and electronic distribution, among other pharmacophore features) determine ligand conduct in a living organism such as bio-transportation, bioavailability, proteins’ affinity, metabolism, reactivity, and toxicity [34]. This screening procedure was executed to assess the drug-likeness of the molecules using DataWarrior to evaluate the physicochemical characteristics and subsequently comparing them to those of acarbose using Lipinski’s rule of five. Additionally, these molecules were evaluated as to whether they were mutagenic, tumorigenic, irritant, or whether they had reproductive effects.

As shown in Table 2, some of the compounds are predicted to have no toxic properties. Compounds **7** and **8** are shown to be mutagenic and tumorigenic. Compounds **1**, **4**, and **9** are predicted to have a reproductive effect with compound **9** further being shown to be an irritant. All the other compounds are predicted as being non-mutagenic, non-irritant, non-tumorigenic, and have no reproductive effects.

The acceptable TPSA values range from 0 to 140 Å as molecules with a greater value tend to be poor at permeating cell membranes [33]. The compounds **1**–**11** obeyed this rule. However, acarbose had a TPSA value > 140 Å. Log P, MW, and TPSA values indicate that the compounds have good membrane permeability and oral bioavailability. Indeed, hydrophobicity, membrane permeability, and drug molecule bioavailability are all affected by these variables, in addition to HBA and HBD. Acceptable RB values also reflect good compound intestinal permeation and oral absorption. TPSA is also useful in determining drug transport and biodistribution behavior [35].

The hydrophobicity of a molecule is directly proportional to log P. The LogP values of between −2 and 6.5 indicate that the molecule is sufficiently hydrophobic and will therefore permeate through cellular membranes as there is an appropriate balance of permeability and solubility [36,37]. The LogP values (see Table 2) indicate that these molecules are hydrophobic and will therefore have a higher affinity for the organic phase over water. The compounds have optimum logP values within the range (0.9–4.3).

To determine the structural skeleton similarity, the compounds were subjected to DataWarrior software analysis, which was used to enumerate and classify eight structural skeleton variables and look for similarities (Table 2). We evaluated the count of intramolecular rotatable bonds to determine the compounds’ flexibility and discovered that only compound **7** had one rotatable bond while all others were composed of two–six rotatable bonds and acarbose with the highest number (9). Small molecules’ electronegative atoms (N, O, S, F, Cl) play a very important role in the formation of hydrogen bonds with protein amino acid residues; seven of these atoms were counted in compound **7**, while the other compounds had two–six electronegative atoms. The reference drug acarbose had 19 electronegative atoms.

We calculated the number of H-bond acceptors and donors to establish the type of electronegative atoms. Six of the molecules presented 1–2 H-donor atoms. In contrast, H-acceptor atoms were found in all the compounds, which contained two–seven H-acceptor atoms. The ring closures, most of which consist of carbon atoms engaged in electrostatic and hydrophobic interactions, were tallied, and all compounds had one to six rings, including acarbose with four. We considered both carbo- and heteroring closures to establish the form of the ring closures. Additionally, we discovered all compounds contained one–three3 carbo-rings. Unlike carbo-rings, 1–4 heterorings were found in 10 compounds, including acarbose. Lastly, we calculated the number of aromatic carbon atoms, and all but acarbose had 6–18 aromatic atoms. We discovered no inverse or direct correlation between the factors we studied and the compounds’ binding affinities after characterizing and analyzing them.

### 3.3. ADME/Tox Prediction

The goal of the in silico absorption, distribution, metabolism, and excretion (ADME) profiling is to reduce high, late drug attrition during drug development and optimizing testing by focusing only on the most promising candidates. The predicted values of ADME for compounds **1**–**11** and the reference, acarbose, are presented in Table 3. ADME/Tox properties are related to pharmacokinetic (absorption, distribution, metabolism, excretion) and pharmacodynamic (drug efficacy and toxicity) characteristics of drugs. The in silico prediction of these properties is important, particularly in the optimization of drug leads or new drug molecules. ADME properties affect drug membrane permeation, oral bioavailability, and drug metabolism [35]. In this study, the eleven compounds were predicted to have high gastrointestinal absorption with values ranging from 93.66 to 97.93%. However, the reference acarbose has very poor intestinal absorption. The data on human intestinal absorption are the total amount of drug absorbed and bioavailable as a percentage of aggregate excretion in feces, bile, and urine [38]. It is worth noting that, because acarbose is designed to work in the gut, a low level of oral bioavailability does seem to be therapeutically preferable.

**Figure 3:**
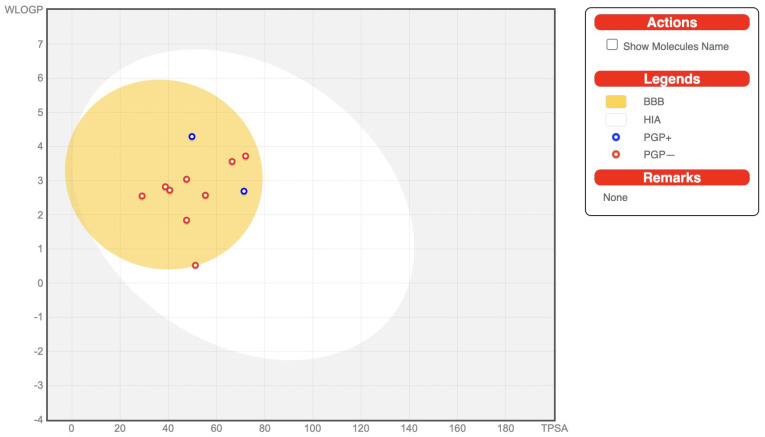
BOILED-Egg plot for compounds **1**–**11**.

These compounds including the reference were predicted to have no permeation across the blood–brain barrier (BBB). The BBB penetration is calculated as the proportion of drug concentration levels in the brain and blood [39]. The predicted plasma protein binding (PPB) values indicated that the molecules are variedly bound to the protein plasma with values ranging from 40.15–90.55%. However, acarbose is bound to a low extent to the plasma protein. Only the unbound drug is typically available for permeation across cell membranes in order to reach the pharmacological target and elicit the desired activity [40]. Therefore, this property can not be emphasized enough. The Caco-2 (human colon carcinoma cell line) penetrability for the estimation of orally administered drug absorption of compounds **1**–**11** ranged from 24.39 to 57.03% with the reference, acarbose, showing no permeation through the Caco-2 cell membrane. This implied that these compounds can be administered orally, with considerable permeation across cell membranes. The transdermal efficacy of these molecules as demonstrated by their skin permeability including the reference indicates that they cannot penetrate through the skin except for compound **2**. It is reported that drugs with logKp values higher than −2.5 cm/h will not penetrate through the skin with ease [41]. The BOILED-Egg plot between WLOGP and TPSA to predict gastrointestinal absorption and brain penetration of the selected molecules is shown in Figure 3. It can be seen from the plot that the molecules are predicted to possess BBB permeant properties and considerable GI absorption.

The metabolism of a drug is another important pharmacokinetic property that should be evaluated during drug development. Cytochrome P450, a family of isozymes, is one of the enzymes taking part in the liver metabolism and biotransformation of drugs. The metabolism of drugs by the cytochrome P450 system is a vital factor in drug interactions that can result in toxicities and a decrease in pharmacological activity. Therefore, determining if the drug is a substrate, inducer, or inhibitor of cytochrome P450 is important. There are various cytochrome P450 isozymes such as CYP1A2, CYP2C19, CYP2C9, CYP2D6, CYP2E1, and CYP3A4 that are involved in drug metabolism [42]. According to the ADME prediction using SwissADME, ligands **1**, **2**, and **4** are non-inhibitors for CYP2C19, CYP2C9, CYP2D6, and CYP3A4. Compounds **5**–**11** are however inhibitors of CYP2C9 and CYP3A4. Compounds **5**, **6**, **9**–**11** are also inhibitors of CYP2C19. The reference acarbose inhibits CYP3A4 and CYP2D6. The ADME properties of these compounds show satisfactory drug qualities.

To ensure that the compounds do not harm human cells and organs, in silico toxicity predictions were executed. This prediction is essential in the early stages of drug discovery because many drug candidates fail in clinical trials due to toxicity. The prediction of toxicological properties of the ligands was performed using the ProTox-II webserver (see Table 3). The ProTox-II predicts the oral toxicity of compounds based on 2D molecular graph similarities with 33,000 compounds and their associated LD_50_ values. Other properties predicted include toxicological endpoints (immunotoxicity, carcinogenicity, and cytotoxicity) and organ toxicity (hepatotoxicity) [43]. Compounds **1**–**11** were predicted to have LD_50_ values of 1000, 2031, 1990, 760, 600, 1000, 2000, 2000, 3919, 720, and 1500 mg/kg, respectively. Acarbose was predicted to have an LD_50_ value of 24,000 mg/kg. The ADMET properties indicate that these molecules pass the adsorption, distribution, metabolism, excretion, and toxicity parameters having shown acceptable bioavailability scores and are orally safe, properties that mostly determine the success of a drug lead [44].

### 3.4. Molecular Docking

The target of the isolation of any natural product is to discover biologically potent therapeutic molecules. The isolation of secondary metabolites and screening of their biological activity, on the other hand, is a tedious, lengthy, and expensive endeavor that can be eased by the use of in silico methods. Molecular docking, a well-documented and powerful in silico approach, can help sieve out inactive compounds. It estimates the modes of interaction between optimized conformations of various compounds and a protein structure. It also helps to predict the probable mode of action of observed biological activity. Given the possibility of this viewpoint, we elaborate on comprehensive molecular docking analyses of isolated compounds from *Zanthoxylum chalybeum* Engl. which have been demonstrated to have varied inhibitions against α-glucosidase and α-amylase. We investigate the binding modes between both the targeted proteins and the ligands using the MOE software.

**Figure 4:**
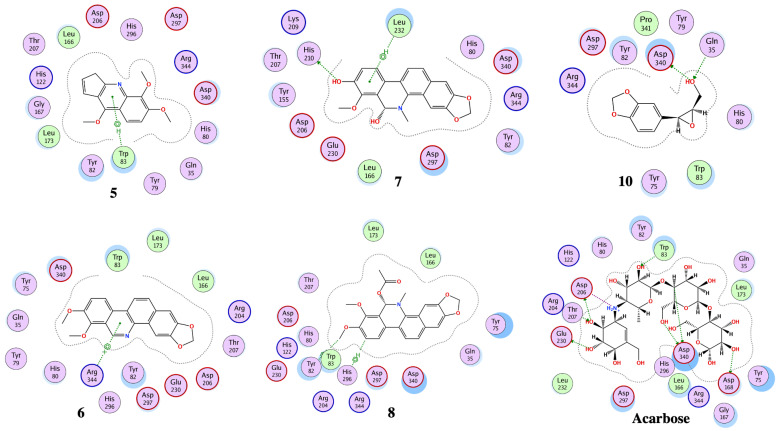
The 2D interactions of Skimmianine (**5**), Norchelerythrine (**6**), 6-Acetonyldihydrochelerythrine (**7**), and 6-Hydroxy-N-methyldecarine (**8**) alkaloids, 2,3-Epoxy-6,7-methylenedioxyconiferol (**10**), and the reference acarbose with α-amylase enzyme (PDB ID: 7TAA).

**Figure 5:**
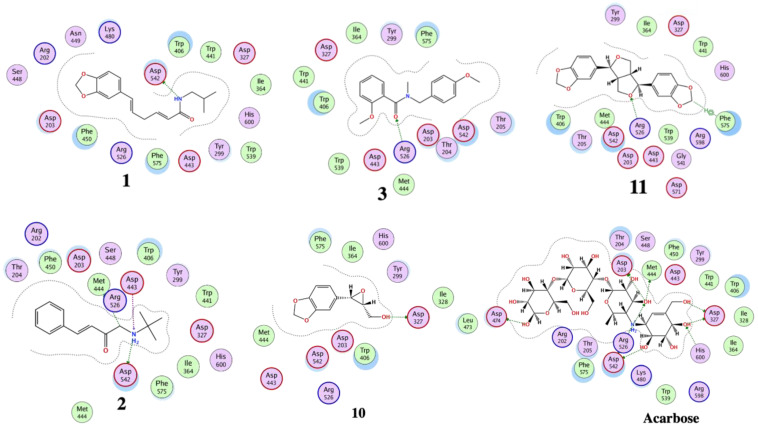
The 2D interactions of chaylbemide A (**1**), chalybeate B (**2**), and chalybemide C (**3**), 2,3-Epoxy-6,7-methylenedioxyconiferol (**10**), sesame (**11**), and the reference acarbose with α-glucosidase enzyme (PDB ID: 2QMJ).

**Figure 6:**
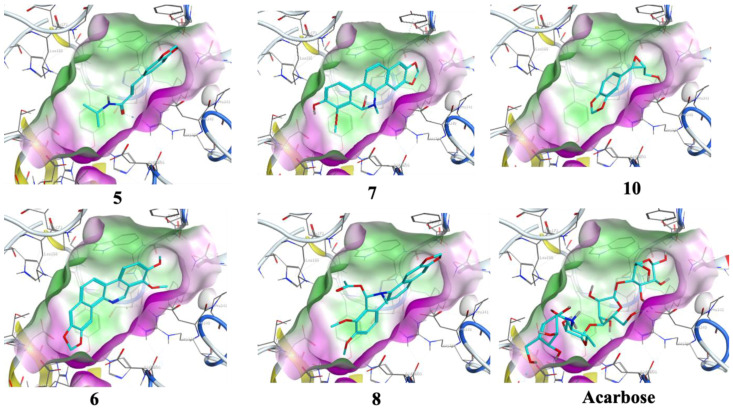
Docking conformation with the highest score conformers for Skimmianine (**5**), Norchelerythrine (**6**), 6-Acetonyldihydrochelerythrine (**7**), and 6-Hydroxy-N-methyldecarine (**8**) alkaloids, 2,3-Epoxy-6,7-methylenedioxyconiferol (**10**), and the reference acarbose (carbon atoms in cyan) in the binding site of α-amylase (ID: 7TAA). Lipophilic areas on the molecular surface are colored green, while hydrophilic areas are colored purple.

**Figure 7:**
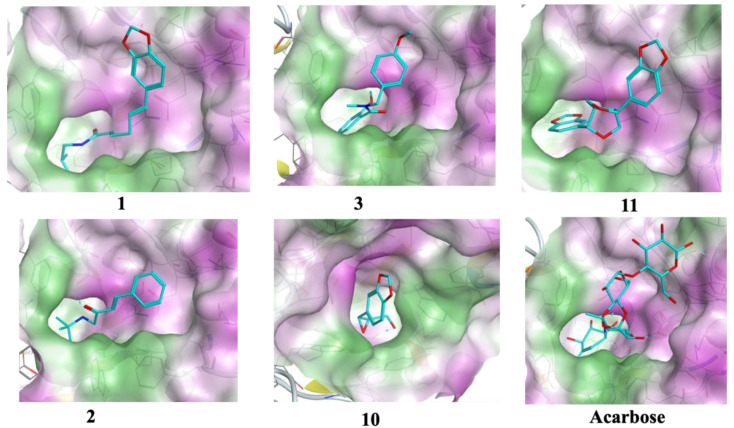
Docking conformation with the highest score conformers for chaylbemide A (**1**), chalybeate B (**2**) and chalybemide C (**3**), 2,3-Epoxy-6,7-methylenedioxyconiferol (**10**), sesame (**11**), and the reference acarbose (carbon atoms in cyan) in the binding site of α-glucosidase (ID: 2QMJ. Lipophilic areas on the molecular surface are colored green, while hydrophilic areas are colored purple.

The optimized structures (**1**–**11**) of the active compounds from *Zanthoxylum chalybeum* Engl. were docked into the active site of the α-glucosidase protein (N-terminal glucoamylase PDB ID: 2QMJ). These compounds were discovered to have good docking scores (−8 to −13 kcal/mol) and binding interactions with the amino residues Asp203, Asp327, Asp542, Arg526, and His600 (Table 4, Figure 4, Figure 5, Figure 6 and Figure 7, and Figures S25–S28 (Supplementary Materials)). Human α-glucosidase has a structure similar to that of human glycoside hydrolase family GH311 homologues, maltase glucoamylase, and sucrase-isomaltase. A trefoil at the N-terminus, the Type-P domain is linked to the β-sheet domain. The catalytic (β/α)8 barrel then follows with its two inserts β3 (insert I) and β4 (insert II). This is then proceeded by proximal and distal β-sheet domains at the C-terminus. The narrow substrate-binding pocket is formed by a loop from the N-terminal-sheet domain and inserts I and II and is located near the C-terminal ends of the catalytic (β/α)8 domain’s β-strands. Asp518 function as the catalytic nucleophile while Asp616 is the acid/base catalyst [45].

Compounds **1** and **3** which showed noncompetitive inhibition on both enzymes, and compound 2 with uncompetitive inhibition and noncompetitive inhibition on α-glucosidase and α-amylase respectively, demonstrated binding affinities of between −13.8 and −9.3 (kcal/mol) against α-amylase and α-glucosidase. Compound 1 formed conventional hydrogen interactions with α-amylase interface residues ASP 297 and ARG 344 (see Table 4 and Figure 5 and Figure 7). In addition, it formed hydrogen bond interactions with ASP 542 residues in the case of α-glucosidase. On the other hand, compound 2 formed hydrogen bond interactions with ASP 340 and ARG 344 in addition to the ionic bond with ASP 340 with α-amylase. On analysis of this compound’s interactions with α-glucosidase, it was found that it forms hydrogen bond interactions with MET 444 and ASP 542 residues in addition to ionic interactions with ASP 443 and ASP 542 residues. Conversely, compound **3** formed hydrophobic interactions of the types H-π and π-π, with HIS 296 and TYR 82 residues of the α-amylase, respectively. Analysis of compound 3 in complex with α-glucosidase revealed the presence of a hydrogen bond interaction with ARG 526 residue.

Compound **4**, which had noncompetitive inhibition on both α-amylase and α-glucosidase but with *K_i_* values significantly higher than those of the reference, formed a hydrogen bond-type interaction with the ASP 340 residue of the α-amylase and a binding affinity of −9.4 kcal/mol. Docking of this compound on the binding site of α-glucosidase, indicated a binding affinity of −9.0 kcal/mol and analysis of the complex interactions revealed the presence of hydrogen bond-type interactions with MET 444 and ASP 542 residues in addition to H-π-type hydrophobic interactions with the PHE 575 residue.

Compounds **5–8** were established to have mixed inhibitions on both enzymes and from the docking simulations registered binding affinities of between −9.5 and −13.8 kcal/mol. Compound **5** formed a hydrophobic interaction of the π-H-type with the TRP 83 residue of the α-amylase. It also showed a hydrogen bond-type as well as hydrophobic interactions of the type π–H with ASP 542 and TRP 406 residues of the α-glucosidase. Compound **6** formed conventional hydrophobic interactions of the π-cation type with the α-amylase interface residue ARG 344. Compound **7** formed conventional hydrogen interactions and hydrophobic π-H-type interactions with α-amylase interface residues HIS 210 and LEU 232, respectively. In complex with α-glucosidase, it formed hydrogen bond interactions with ASP 443 and ASP 542 residues. Docking compound **8** onto the binding pocket of α-amylase and α-glucosidase showed hydrophobic interactions of the H-π-type with α-amylase interface residues HIS 296 and TYR 82 observed. With THR 204 residues of α-glucosidase, it formed π-H hydrophobic interactions.

Compounds **9** and **11** showed uncompetitive inhibition while compound **10** had competitive inhibition on both proteins. Compound **9** demonstrated binding affinities of −11.4 and −10.3 kcal/mol against α-amylase and α-glucosidase, respectively. It displayed conventional hydrogen interactions and hydrophobic π-H-type interactions with α-amylase interface residues ASP, 340, GLN 35, and TYR 79, and HIS 296, respectively. On analysis of its interaction with α-glucosidase, π-H hydrophobic interactions with the ASP 327 residue was observed. With compound 10, binding scores of −10.1 and −10.0 kcal/mol with α-amylase and α-glucosidase were observed, respectively. The α-amylase ASP 340 and GLN 35 residues interacted with compound **10** forming hydrogen bonds. Hydrogen bond interaction was also observed in the case of α-glucosidase with the enzyme ASP 327 residue. Compound **11** formed hydrogen bond-type interactions with ARG 204 and TRP 83 residues of the α-amylase binding pocket. These resulted in a binding score of −12.3 kcal/mol. The molecule formed hydrogen and a π-H type hydrophobic bonds with ARG 526 and PHE 575 residues, respectively, of the α-glucosidase. The binding affinity, in this case, was −11.0 kcal/mol.

Acarbose was used as the control, showing a binding score of −17.6 kcal/mol with α-amylase, and formed ordinally hydrogen bonds with residues ASP 206, GLU 230, ASP 340, ASP 168, ARG 204, and TRP 83. In addition, it formed attractive electrostatic forces with residue ASP 206. On the other hand, with α-glucosidase, a binding affinity of −20.5 kcal/mol was recorded. With ASP 542, ASP 327, ASP 203, MET 444, ASP 474, HαIS 600, and ARG 526 residues of the binding pocket it formed hydrogen bond interactions, in addition to an ionic bond with ASP 542. Apparently, the greater number of H-bond interactions at the binding pocket in acarbose was a result of the higher number of hydroxyl functional groups which formed hydrogen bonds with the active sites’ amino acid residues [46]. In previously reported in silico studies, a number of 1,2-benzothiazine and xanthone derivatives, 8-c-ascorbyl(-)-epigallocatechin, and Voglibose also showed α-glucosidase inhibition through interaction with Asp203, Asp542, and Arg526 pocket residues of the receptor protein [47,48,49,50]. Additionally, reported molecular docking studies of 1, 2-benzothiazine derivative against α-amylase indicated excellent binding affinities with the residues TRP83, ASP340, ARG 204, and GLU 230 [51].

In addition to the control molecules, the eleven ligands formed several other bonds with main amino acid residues as can be seen in Figures S25–S28 which may interrupt the normal physiological functions of these two enzymes. The natural products studied through the molecular docking simulations in this work revealed significant binding energies with the pocket residues for the two proteins. These compounds established networks of different types of interactions (although fewer than the reference) that played a part in the binding affinity of the calculated complexes and therefore affirmed their dual inhibitory activity.

We demonstrate that compounds **1**–**11** are dual inhibitors, effective to both proteins with comparable potency, and the docking studies results show that the compounds bind to the active sites of the receptors with a good binding energy of interactions and RMSD values. Importantly, all the studied compounds had formerly been reported to inhibit α-glucosidase and α-amylase, with IC_50_ values of between 43.22 and 58.21 µM [12]. The dual inhibitors involved in this study, as disruptors of a critical carbohydrate metabolic process, provide a potential starting point for structural optimization in search of more efficacious and highly potent carbohydrate metabolism inhibitors. The newly acquired information on compounds **1**–**11** being some of the few compounds known to have a dual inhibitory activity against α-glucosidase and α-amylase could be helpful in the future to look for inhibitors of such enzyme systems. Furthermore, the current results add an important component to the knowledge of these natural products’ mechanism(s) of action in the management of diabetes. Lastly, it is important to mention that a virtual screening of natural product libraries to yield an assortment of more hits is at the moment being investigated and will be the subject of future communication.

## 4. Conclusions

In conclusion, this study aimed to find a potential duo inhibitor for α-amylase and α-glucosidase from the phytochemicals of the medicinal plant *Zanthoxylum chalybeum* Engl., examined their mode of interaction with protein residues of the binding pocket, and profiled their ADMET and Toxicity properties in silico. In comparison to the standard control (acarbose), the compounds exhibited remarkable inhibition constants (Ki), binding affinities and strong interactions with crucial pocket amino acid residues of the α-amylase and α-glucosidase proteins. The results of this study indicate that these molecules have the potential to be antidiabetic drugs by inhibiting α-amylase and α-glucosidase which are responsible for the metabolism of carbohydrates into absorbable simple sugars. Through inhibition of these enzymes, the absorption of dietary sugars and the succeeding postprandial upsurge in blood glucose and insulin levels is limited. However, more experimental studies are required to confirm the antidiabetic activity of these compounds in vivo.

## Figures and Tables

**Table 1:** Inhibition modes and inhibition constant (*K_i_*) values obtained from Lineweaver–Burk plots and Dixon Plots, respectively, for metabolites of *Z. chalybeum* against α-glucosidase and α-amylase inhibition in comparison to the reference acarbose inhibitions.

Compound	α-Amylase			α-Glucosidase		
	Inhibition Mode	*K_i_* (mM)	R^2^	Inhibition Mode	*K_i_* (mM)	R^2^
**1**	Non-competitive	13.36 ± 2.43 **	0.9345	Non-competitive	20.95 ± 2.14	0.9824
**2**	Non-competitive	11.05 ± 0.58 **	0.9424	Uncompetitive	44.58 ± 1.65	0.9207
**3**	Non-competitive	14.83 ± 0.50 **	0.9381	Non-competitive	17.56 ± 0.24	0.9824
**4**	Non-competitive	26.69 ± 2.13 **	0.9899	Non-competitive	34.73 ± 0.79 **	0.9198
**5**	Mixed	2.74 ± 0.06	0.9572	Mixed	7.64 ± 0.02 **	0.9544
**6**	Mixed	7.57 ± 0.59	0.9527	Mixed	7.68 ± 0.04 **	0.9578
**7**	Mixed	3.34 ± 0.03	0.9978	Mixed	4.73 ± 0.10 **	0.9966
**8**	Mixed	3.10 ± 0.20	0.9753	Mixed	9.17 ± 0.10 **	0.9913
**9**	Uncompetitive	26.28 ± 1.47 **	0.9619	Uncompetitive	20.62 ± 1.94	0.9929
**10**	Competitive	5.54 ± 1.02	0.9850	Competitive	17.21 ± 0.16	0.8692
**11**	Non-competitive	12.53 ± 1.957 **	0.9552	non-competitive	24.33 ± 1.93	0.8748
Acarbose	Competitive	6.14 ± 0.01	0.9606	Competitive	22.40 ± 1.23	0.8387

** values are significantly different from the standard inhibitor (acarbose) Inhibition constant (*K_i_*) based on Tukey HSD/Tukey Kramer post-analysis of one-way analysis of variance replicated Ki values. Least significant difference was considered at *p* < 0.05 and the coefficient of determination (R^2^) was obtained as the average of the regression curves from Dixon plots of individual experiments.

**Table 2:** Physicochemical properties of compounds **1**–**12**.

Molecule	MW ^a^	LogP ^b^	HBA ^c^	HBD ^d^	RB ^e^	Electronegative Atoms	Rings Closures	Carbo-rings	Hetero-rings	Aromatic Atoms	TPSA ^f^ (Å)	Mutagenic	Tumorigenic	Reproductive Effect	Irritant
**1**	287.358	3.7	4	1	6	2	1	1	0	6	47.6	none	none	high	none
**2**	218.319	1.1	2	1	5	4	2	1	1	6	33.7	none	none	none	none
**3**	285.342	2.6	4	0	5	4	2	1	1	12	38.8	none	none	none	none
**4**	233.266	2.3	4	1	3	4	2	1	2	6	47.6	none	none	high	none
**5**	257.288	2.5	4	0	3	4	3	1	3	10	40.6	none	high	none	none
**6**	333.342	4.3	5	0	2	4	3	2	0	18	49.8	low	none	none	none
**7**	351.357	3.3	6	2	1	5	5	2	1	16	71.4	high	high	none	none
**8**	407.421	4.1	7	0	4	5	3	2	1	16	66.5	high	high	none	none
**9**	326.347	3.5	5	2	5	6	5	2	4	15	72.1	none	none	high	high
**10**	194.185	0.9	4	1	2	6	6	3	2	6	51.2	low	none	none	none
**11**	354.357	3.2	6	0	2	7	5	3	2	12	55.4	none	none	none	none
**12**	646.613	−8.4	19	14	9	19	4	3	2	0	325.8	none	none	none	none

^a^: Molecular weight; ^b^: Partition coefficient between n-octanol and water; ^c^: Hydrogen bond acceptor; ^d^: Hydrogen bond donor; ^e^: Rotatable bond; ^f^: Topological polar surface area (Å).

**Table 3:** ADME/Tox properties for compounds **1**–**11** and the reference, acarbose.

Property\Compound	BBB	HIA (%)	Plasma Protein Binding (%)	CYP3A4 Inhibitor	CYP2C9 Inhibitor	CYP2D6 Inhibitor	CYP2C19 Inhibitor	Skin Permeability (cm/h)	Caco2	Hepatotoxicity	Carcinogenicity	Immunotoxicity	Cytotoxicity	Toxicity Class
**1**	1.13	95.34	87.74	non	non	non	non	−2.86	44.84	Inactive	Inactive	Active	Inactive	4
**2**	0.79	96.22	40.15	non	non	yes	non	−1.03	54.93	Inactive	Inactive	Inactive	Inactive	5
**3**	0.17	98.11	87.07	non	non	non	non	−2.92	54.35	Inactive	Inactive	Inactive	Inactive	4
**4**	0.14	94.94	53.54	non	non	non	non	−3.43	38.10	Inactive	Inactive	Active	Inactive	4
**5**	2.68	97.93	90.19	yes	yes	non	yes	−3.74	56.83	Active	Inactive	Active	Inactive	4
**6**	0.05	97.64	90.55	yes	yes	non	yes	−3.98	52.67	Inactive	Active	Active	Inactive	4
**7**	0.20	94.62	87.76	yes	yes	non	non	−4.02	21.94	Inactive	Inactive	Active	Active	4
**8**	0.02	97.55	89.45	yes	yes	non	non	−3.88	48.50	Inactive	Active	Active	Active	4
**9**	0.28	94.23	88.29	yes	yes	non	yes	−2.92	30.85	Inactive	Inactive	Active	Inactive	5
**10**	0.58	93.66	45.96	yes	yes	non	yes	−3.97	24.39	Inactive	Active	Inactive	Inactive	3
**11**	0.05	97.12	83.12	yes	yes	non	yes	−4.42	57.03	Inactive	Active	Active	Inactive	3
Acarbose	0.03	0.00	31.61	yes	non	yes	non	−5.19	0.81	Active	Inactive	Active	Inactive	6

**Table 4:** The interactions and binding affinities of compounds **1**–**11** and acarbose with α-amylase enzyme (7TAA) and α-glucosidase enzyme (2QMJ) residues discovered during complex structure visualization.

	α-Amylase				α-Glucosidase			
Compound No.	Docking Score (kcal/mol)	RMSD Value (Å)	Binding Residues	Interaction	Docking Score (kcal/mol)	RMSD Value (Å)	Binding Residues	Interaction
**1**	−11.2	1.5	ASP 297 ARG 344	H-donor H-acceptor	−9.3	2.0	ASP 542	H-donor
**2**	-13.8	0.8	ASP 340 ARG 344 ASP 340	H-donor H-acceptor Ionic	-12.6	1.8	MET 444 ASP 542 ASP 443 ASP 542	H-donor H-donor Ionic ionic
**3**	−10.1	0.8	HIS 296 TYR 82	H-π π-π	−10.6	0.9	ARG 526	H-acceptor
**4**	−9.4	0.8	ASP 340	H-donor	−9.0	1.2	MET 444 ASP 542 PHE 575	H-donor H-donor H-π
**5**	−10.0	1.9	TRP 83	π-H	−9.5	0.8	ASP 542 TRP 406	H-donor π-H
**6**	−11.9	1.9	ARG	π-cation	−9.8	0.9	-	-
**7**	−10.9	0.8	HIS 210 LEU 232	H-donor π-H	−13.8	0.8	ASP 443 ASP 542	H-donor H-donor
**8**	−10.8	1.3	HIS 296 TYR 82	H-π H-π	−8.0	2.0	THR 204	π-H
**9**	−11.4	1.0	ASP 340 GLN 35 TYR 79 HIS 296	H-donor H-acceptor H-acceptor H-π	−10.3	2.8	ASP 327	H-donor
**10**	−10.1	1.1	ASP 340 GLN 35	H-donor H-acceptor	−10.0	0.6	ASP 327	H-donor
**11**	−12.3	1.2	ARG 204 TRP 83	H-acceptor H-acceptor	−11.0	1.5	ARG 526 PHE 575	H-acceptor H-π
Acarbose	−17.6	1.7	ASP 206 GLU 230 ASP 340 ASP 168 ARG 204 TRP 83 ASP 206	H-donor H-donor H-donor H-donor H-acceptor H-acceptor ionic	−20.5	1.6	ASP 542 ASP 327 ASP 203 MET 444 ASP 474 HIS 600 ARG 526 ASP 542	H-donor H-donor H-donor H-donor H-donor H-acceptor H-acceptor ionic

## Data Availability

Not applicable.

## Supplementary Materials

The following supporting information can be downloaded at: https://www.mdpi.com/article/10.3390/jox13010009/s1, Figures S1–S24: Lineweaver-Burk and Dixon plots for inhibition of α-amylase and α-glucosidase by compounds **1**–**11** and the reference acarbose; Figures S25–S28: 2D and 3D interactions of compounds **1**–**11** and the reference acarbose with α-amylase and α-glucosidase enzymes.
