# Supplementary material for: Inhibition Kinetics and Theoretical Studies on Zanthoxylum chalybeum Engl. Dual Inhibitors of α-Glucosidase and α-Amylase
Source: J Xenobiot. 2023 Feb 21;13(1):102–20. doi: 10.3390/jox13010009 (PMC10059848; doi:10.3390/jox13010009)
Supplement: Supplementary file 1 [file jox-13-00009-s001.zip › supplementary jox-2148689.pdf]

Inhibition kinetics and theoretical studies on *Zanthoxylum chalybeum* Engl. dual inhibitors of α-glucosidase and α-amylase

Njogu M. Kimani[1*] Charles O. Ochieng[2], Mike Don Ogutu[2], Kevin Otieno Yamo[2], Joab Otieno Onyango[3], and Cleydson B. R. Santos[4,5]

[1] Department of Physical Sciences, University of Embu, Embu, Kenya;

[2] Department of Chemistry, Maseno University, Maseno, Kenya;

[3] School of Chemical Sciences and Technology, Technical University of Kenya, Nairobi, Kenya

[4] Graduate Program in Biotechnology and Biodiversity-Network BIONORTE, Federal University of Amapá, Macapá, 68903-419, AP, Brazil

[5] Laboratory of Modeling and Computational Chemistry, Department of Biological and Health Sciences, Federal University of Amapá, Macapá 68902-280, AP, Brazil

S1: Lineweaver-Burk and Dixon plots for inhibition of α-amylase by Chalybemide A (**1**) 2
S2: Lineweaver-Burk and Dixon plot for inhibition of α-glucosidase by Chalybemide A (**1**) 3
S3: Lineweaver-Burk and Dixon plots for inhibition of α-amylase by Chalybemide B (**2**) 4
S4: Lineweaver-Burk and Dixon plots for inhibition of α-glucosidase by Chalybemide B (**2**) 5
S5: Lineweaver-Burk and Dixon plots for inhibition of α-amylase by Chalybemide C (**3**) 6
S6: Lineweaver-Burk and Dixon plots for inhibition of α-glucosidase by Chalybemide C (**3**) **7**
S7: Lineweaver-Burk and Dixon plots for inhibition of α-amylase by fagaramide (**4**) 8
S8: Lineweaver-Burk and Dixon plots for inhibition of α-glucosidase by fagaramide (**4**) 9
S9: Lineweaver-Burk and Dixon plots for inhibition of α-amylase by Skimmianine (**5**) 10
S10: Lineweaver-Burk) and Dixon plots for inhibition of α-glucosidase by Skiamminine (**5**) 11
S11: Lineweaver-Burk and Dixon plots for inhibition of α-amylase by Norchelerythrine (**6**) 12
S12: Lineweaver-Burk and Dixon plots for inhibition of α-glucosidase by Norchelerythrine (**6**) **13**
S13: Lineweaver-Burk and Dixon plots for inhibition of α-amylase by 6-Acetonyldihydrochelerythrine (**7**) 14
S14: Lineweaver-Burk and Dixon plots for inhibition of α-glucosidase by 6-Acetonyldihydrochelerythrine (**7**) 15
S15: Lineweaver-Burk) and Dixon plots for inhibition of α-amylase by 6-hydroxy-N-methyldecarine (**8**) 16
S16: Lineweaver-Burk and Dixon plots for inhibition of α-glucosidase by 6-hydroxy-N-methyldecarine (**8**) 17
S17: Lineweaver-Burk and Dixon plots for inhibition of α-amylase by Ailanthodial (**9**) 18
S18: Lineweaver-Burk) and Dixon plots for inhibition of α-glucosidase by Ailanthoidol (**9**) 19
S19: Lineweaver-Burk and Dixon plots for inhibition of α-amylase by 2,3-epoxy-6,7-methylenedioxyconiferol (**10**) 20
S20: Lineweaver-Burk and Dixon plots for inhibition of α-glucosidase by 2,3-epoxy-6,7-methylenedioxyconiferol (**10**) 21
S21: Lineweaver-Burk and Dixon plots for inhibition of α-amylase by Sesamine (**11**) 22
S22: Lineweaver-Burk) and Dixon plots for inhibition of α-glucosidase by Sesamine (**11**) 23
S23: Lineweaver-Burk and Dixon plots for inhibition of α-amylase by acarbose 24
S24: Lineweaver-Burk and Dixon plots for inhibition of α-glucosidase by Acarbose 25

S25: 2D interactions of compounds 1-11 and the reference Acarbose with α-amylase enzyme (PDB ID: 7TAA). 26
S26: 2D interactions of compounds 1-11 and the reference Acarbose with α-glucosidase enzyme (PDB ID: 2QMJ). 26
S27: 3D interactions of compounds 1-11 and the reference Acarbose with α-amylase enzyme (PDB ID: 7TAA). 27
S28: 3D interactions of compounds 1-11 and the reference Acarbose with α-glucosidase enzyme (PDB ID: 2QMJ). 28

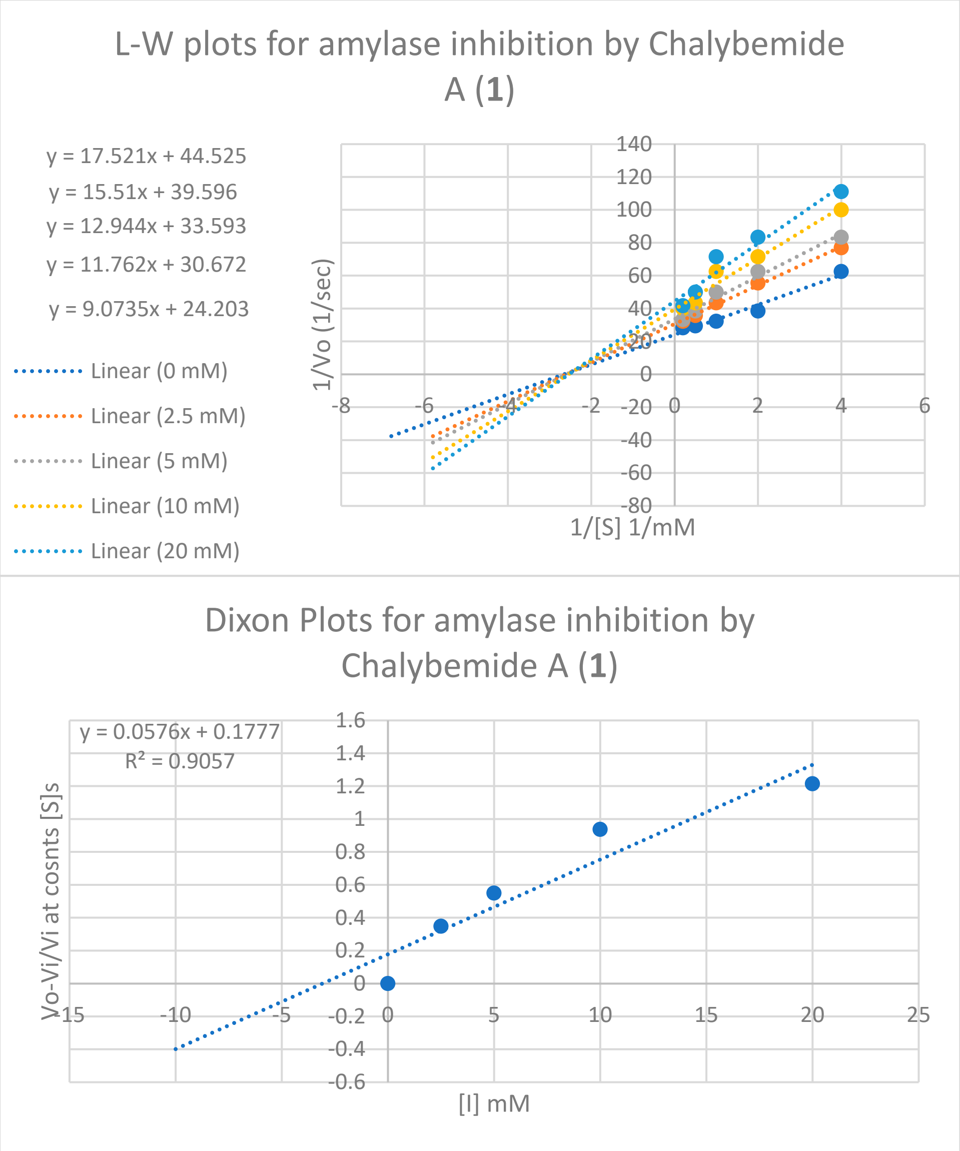

Figure S1: A- Double reciprocal (Lineweaver-Burk) plot for inhibition of *α*-amylase (non-competitive inhibition, Km = 2.59 mM); B- Dixon plot for Chalybemide A (**1**), Ki = **13.36 mM.**

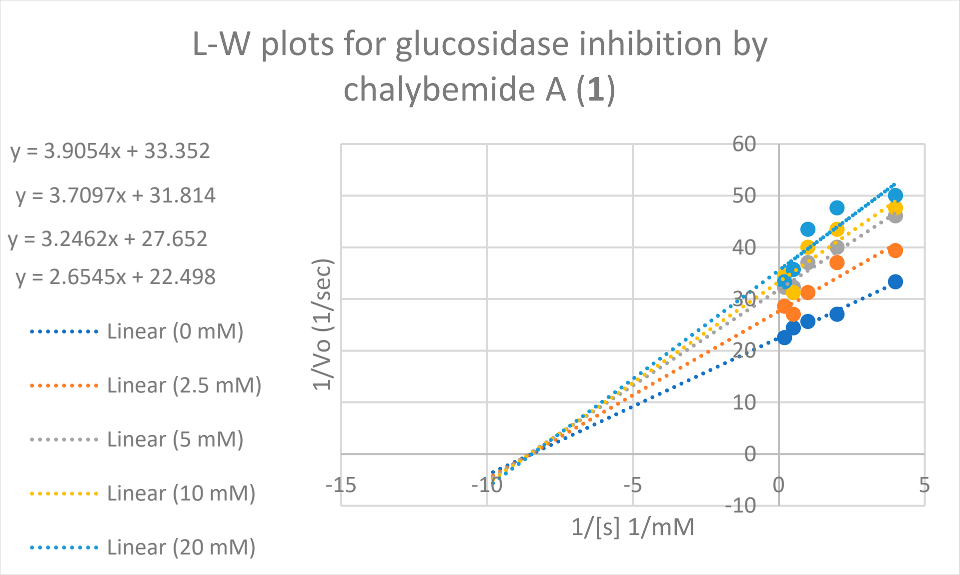

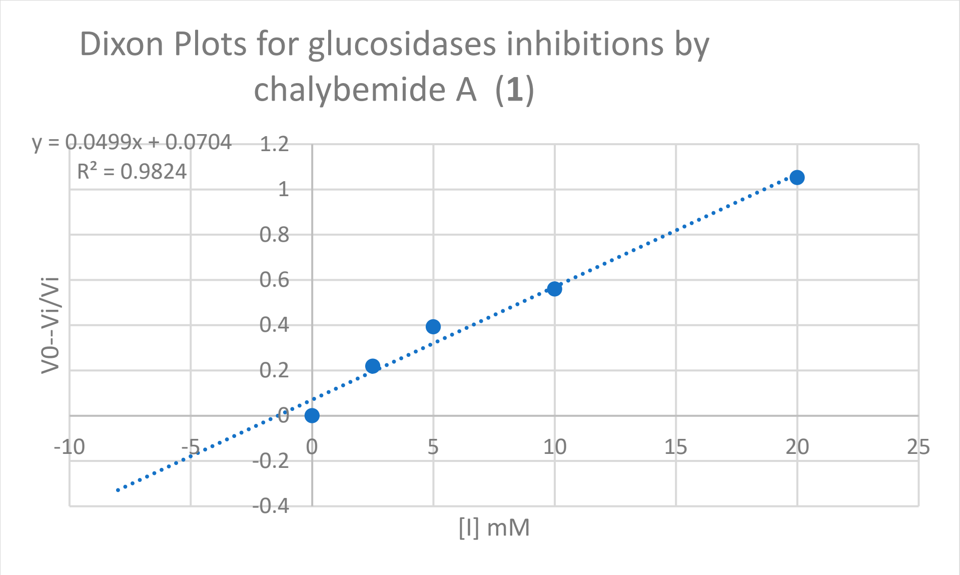

Figure S2: A- Double reciprocal (Lineweaver-Burk) plot for inhibition of α-glucosidase (non-competitive inhibition, Km = 8.53 mM); B- Dixon plot for Chalybemide A (**1**), Ki = **20.95 mM.**

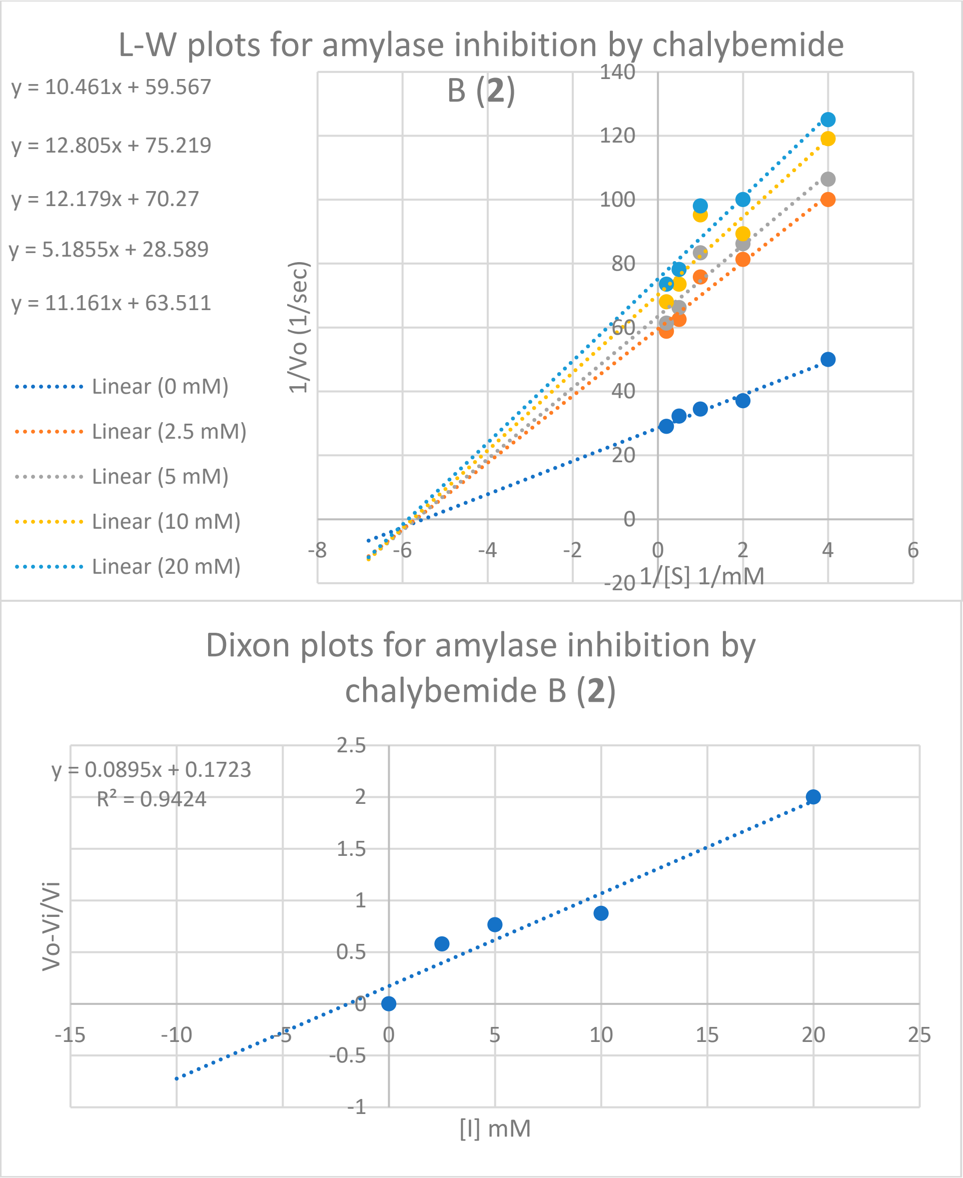

Figure S3: A- Double reciprocal (Lineweaver-Burk) plot for inhibition of α-amylase (non-competitive inhibition, Km = 5.53 mM); B- Dixon plot for Chalybemide B (**2**), Ki = **11.05 mM.**

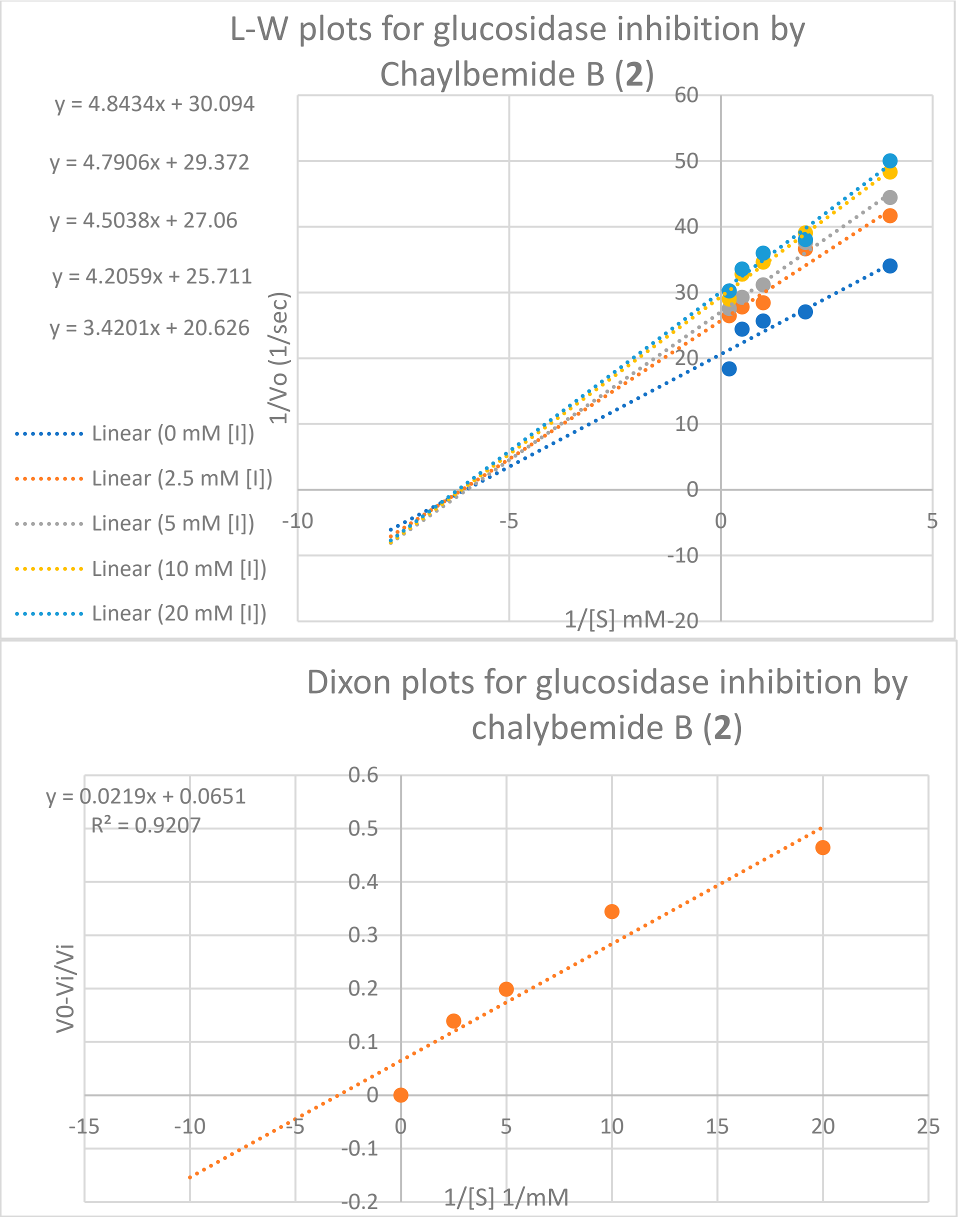

Figure S4: A- Double reciprocal (Lineweaver-Burk) plot for inhibition of α-glucosidase (non-competitive inhibition, Km = 6.10 mM); B- Dixon plot for Chalybemide B (**2**), Ki = **44.58 mM.**

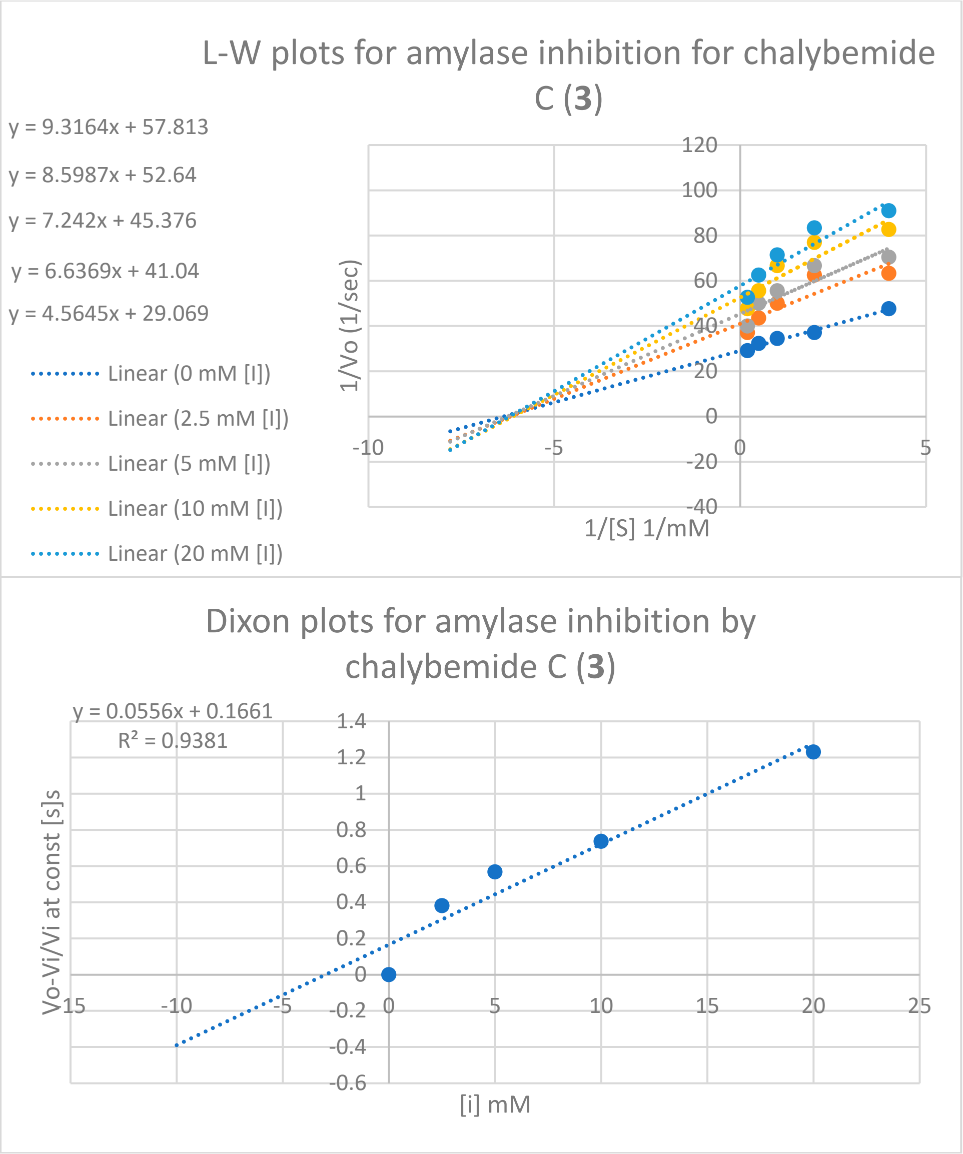

Figure S5: A- Double reciprocal (Lineweaver-Burk) plot for inhibition of α-amylase (non-competitive inhibition, Km = 6.23 mM); B- Dixon plot for Chalybemide C (**3**), Ki = **26.96 mM.**

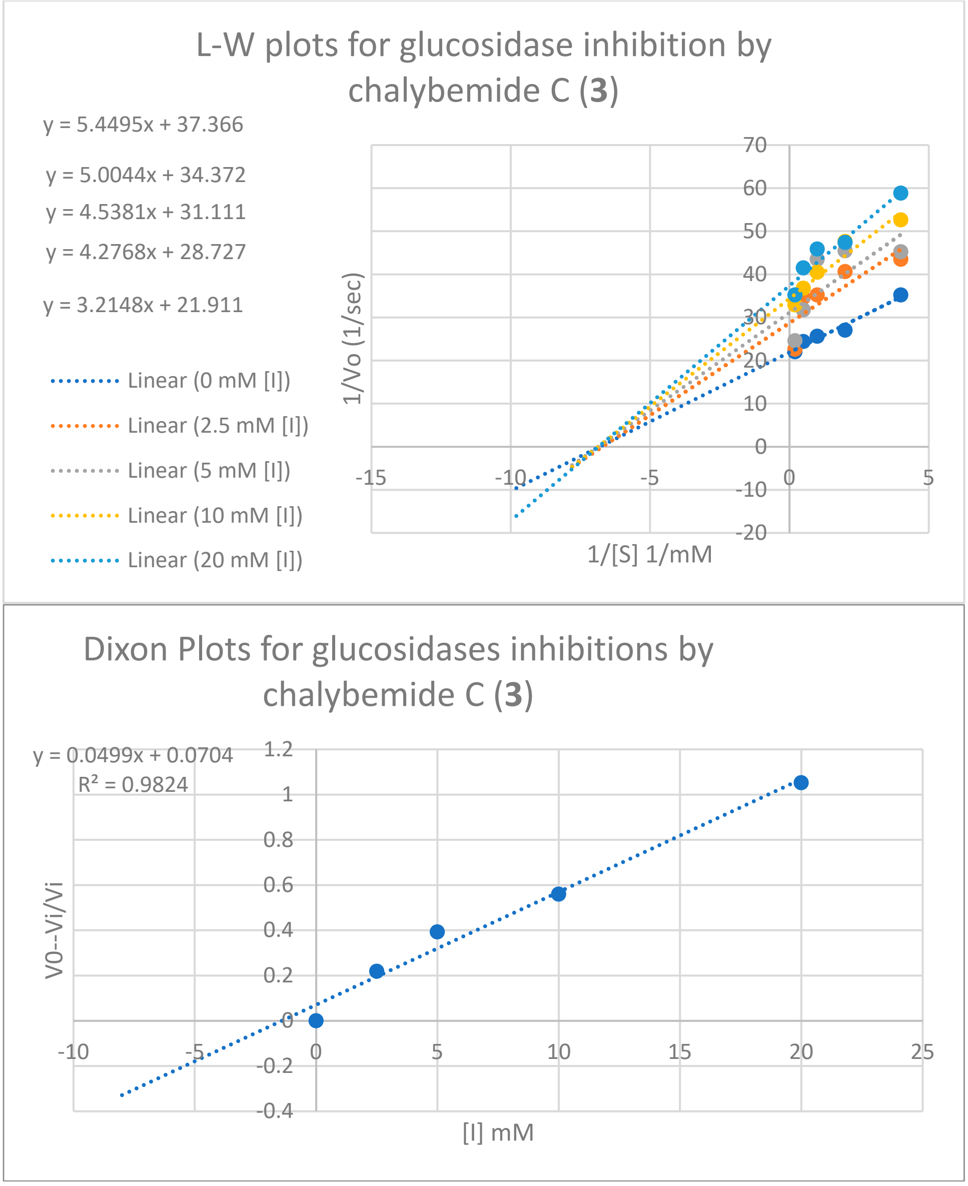

Figure S6: A- Double reciprocal (Lineweaver-Burk) plot for inhibition of *α*-glucosidase (non-competitive inhibition, Km = 6.82 mM); B- Dixon plot for Chalybemide C (**3**), Ki = **34.73 mM.**

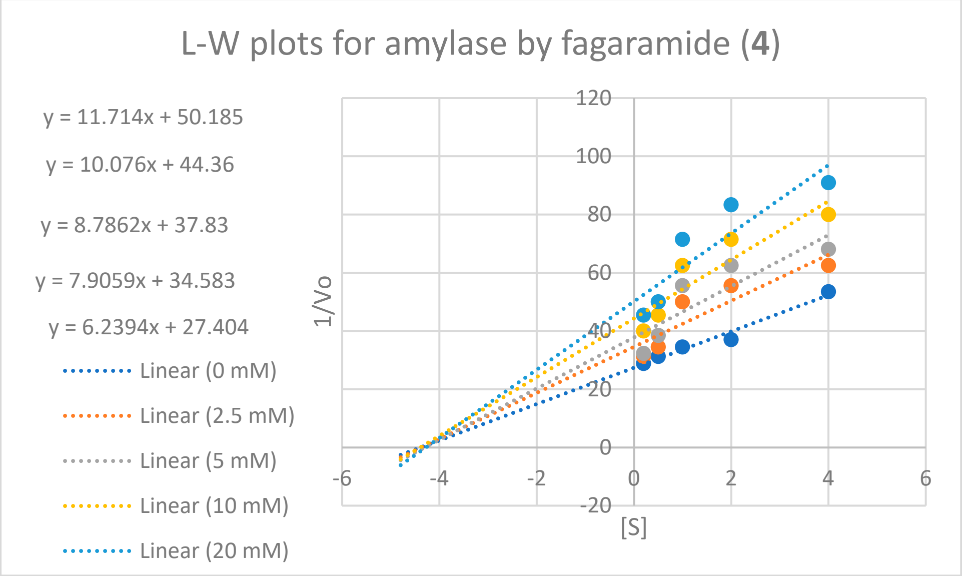

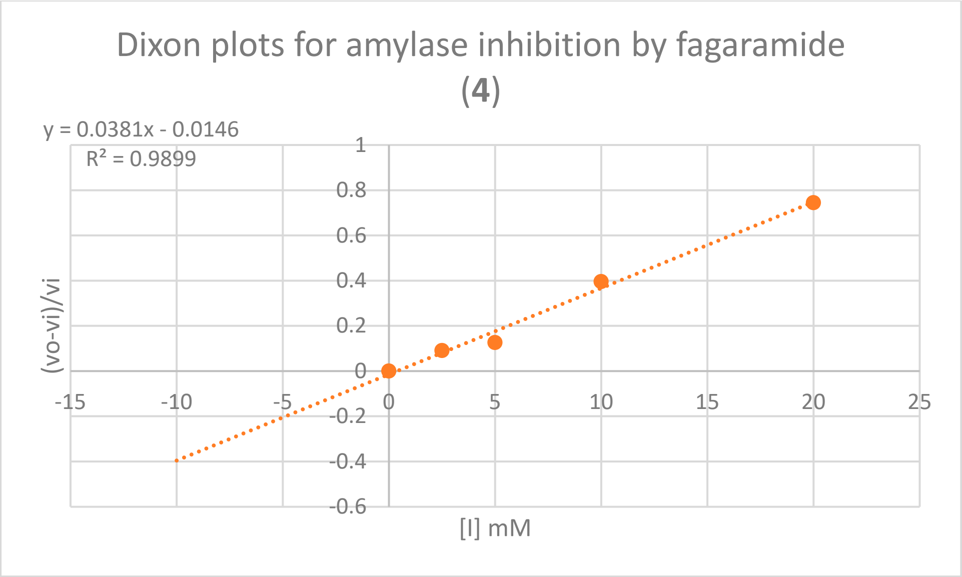

Figure S7: A- Double reciprocal (Lineweaver-Burk) plot for inhibition of α-amylase (non-competitive inhibition, Km = 4.34 mM); B- Dixon plot for fagaramide (**4**), Ki = **26.96 mM.**

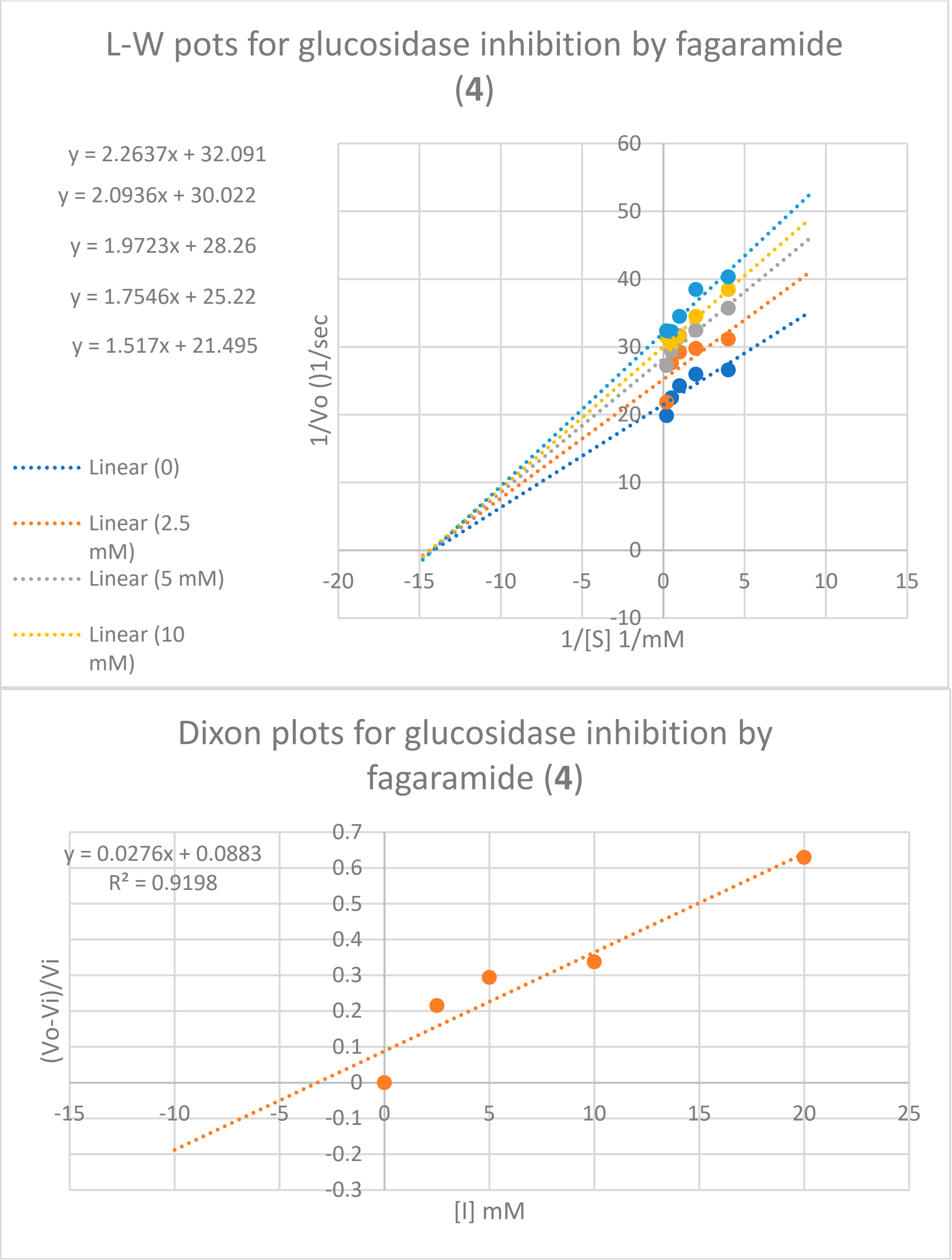

Figure S8: A- Double reciprocal (Lineweaver-Burk) plot for inhibition of α-glucosidase (non-competitive inhibition, Km = 14.25 mM); B- Dixon plot for fagaramide (**4**), Ki = **26.96 mM.**

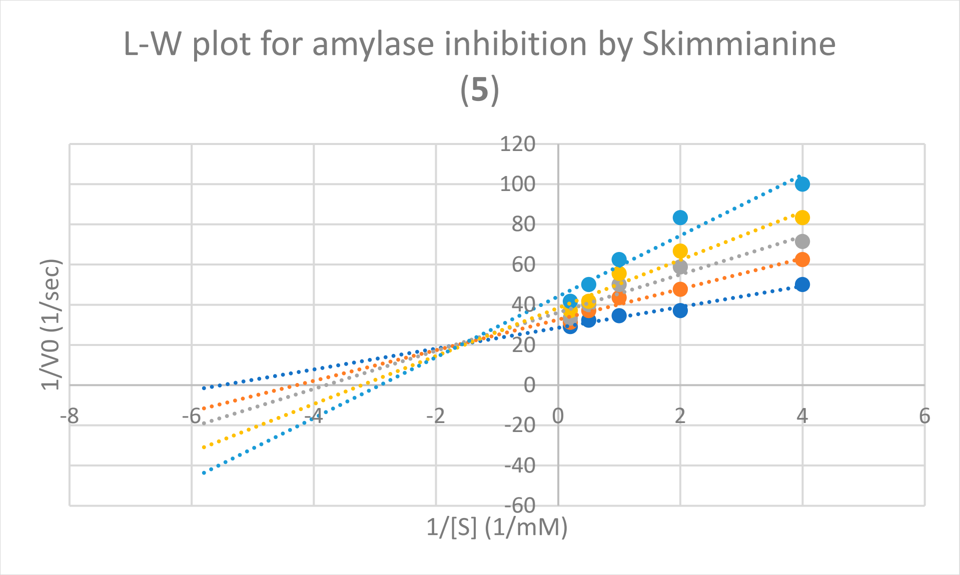

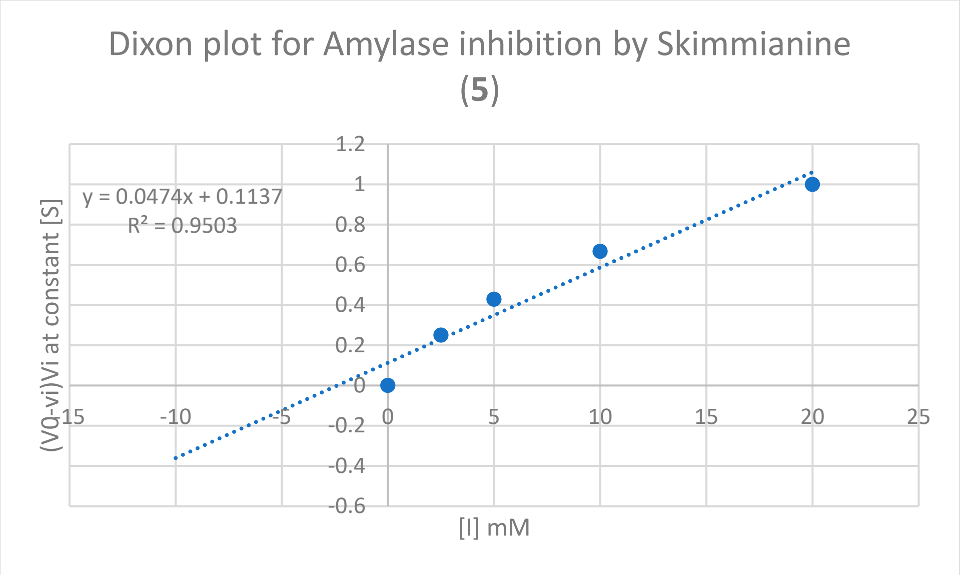

Figure S9: A- Double reciprocal (Lineweaver-Burk) plot for inhibition of α-amylase (mixed inhibition, Varied Km); B- Dixon plot for Skimmianine (**5**), Ki = **2.74 mM.**

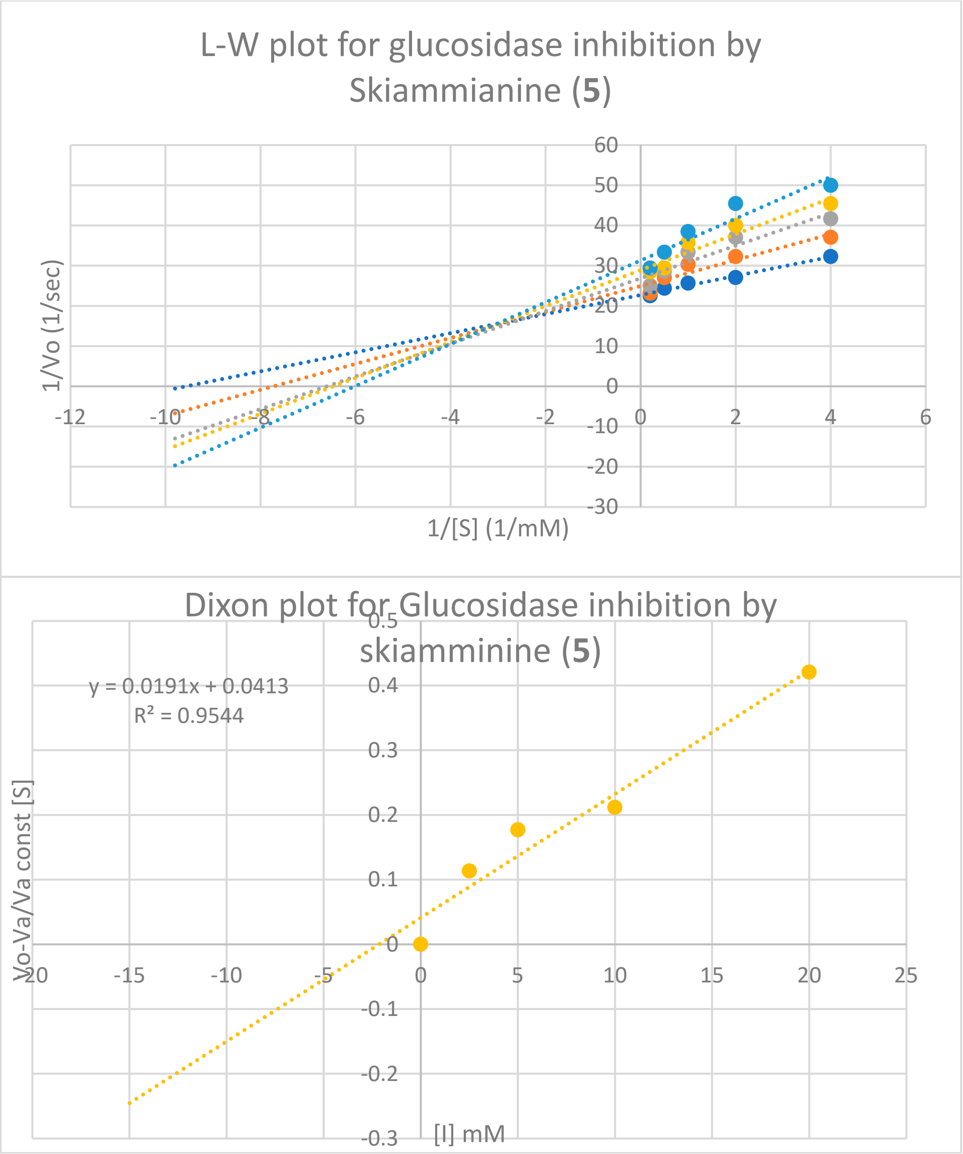

Figure S10: A- Double reciprocal (Lineweaver-Burk) plot for inhibition of α-glucosidase (mixed inhibition, Varied Km); B- Dixon plot for Skiamminine (**5**), Ki = **7.64 mM.**

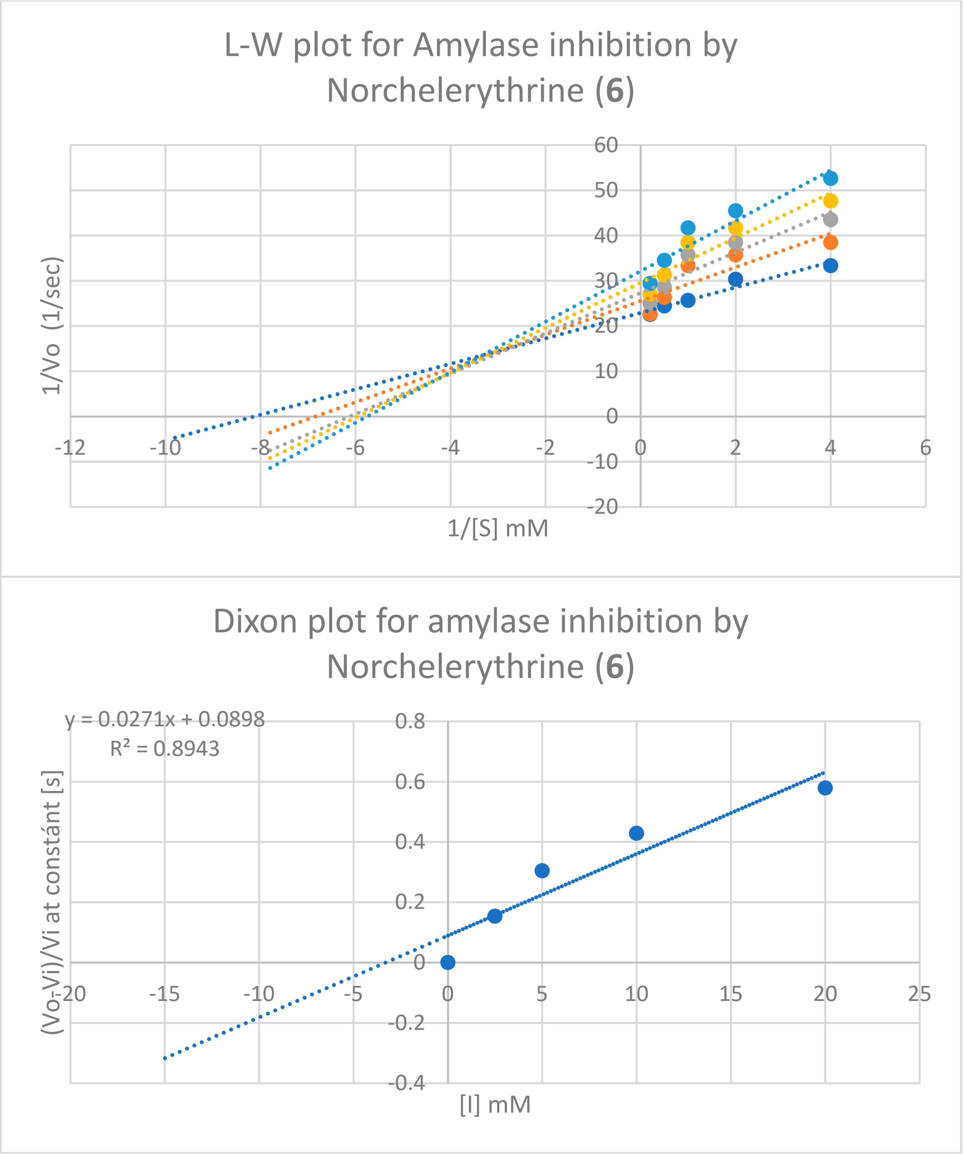

Figure S11: A- Double reciprocal (Lineweaver-Burk) plot for inhibition of α-amylase (mixed inhibition, Varied Km); B- Dixon plot for Norchelerythrine (**6**), Ki = **6.51 mM.**

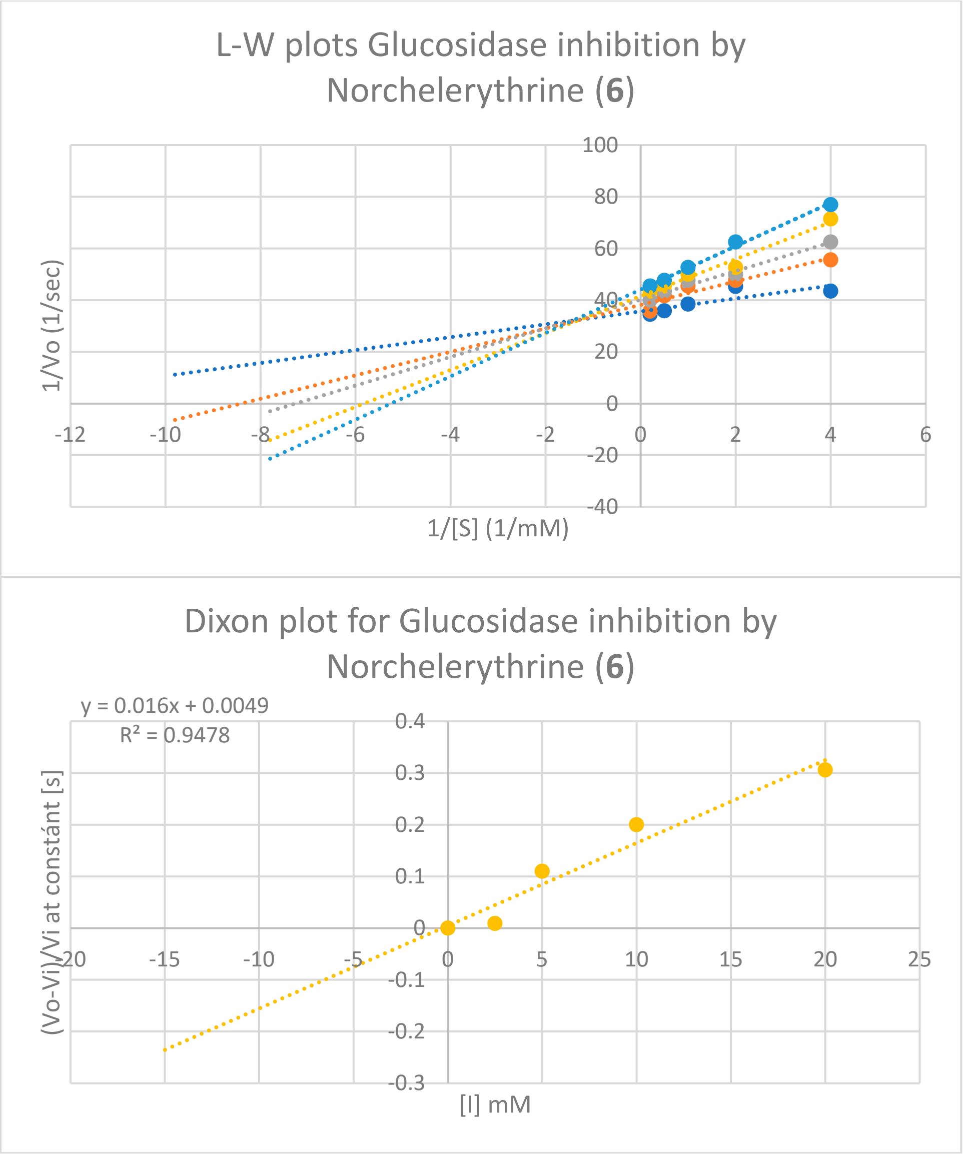

Figure S12: A- Double reciprocal (Lineweaver-Burk) plot for inhibition of α-glucosidase (mixed inhibition, Varied Km); B- Dixon plot for Norchelerythrine (**6**), Ki = **7.68 mM.**

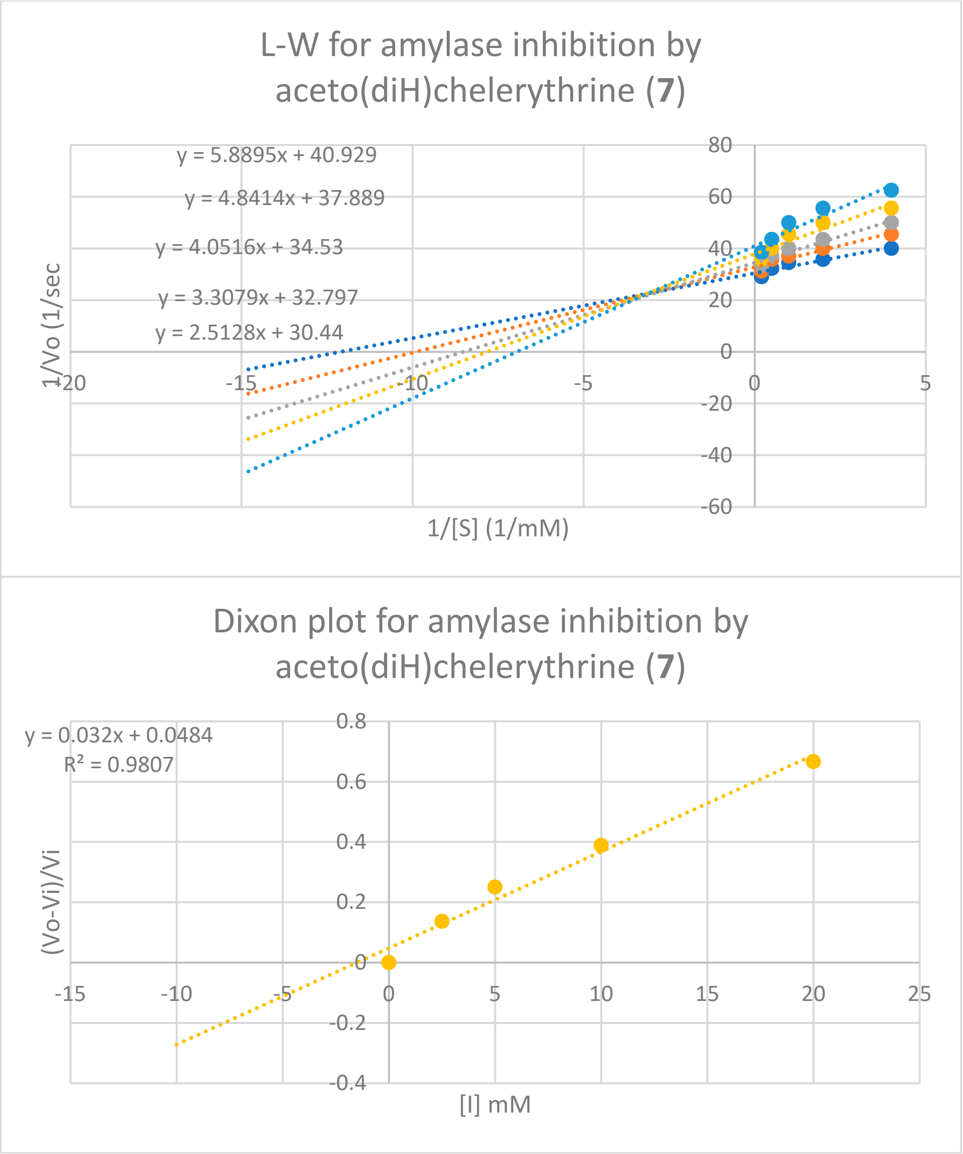

Figure S13: A- Double reciprocal (Lineweaver-Burk) plot for inhibition of α-amylase (mixed inhibition, Varied Km); B- Dixon plot for 6-Acetonyldihydrochelerythrine (**7**), Ki = **3.34 mM.**

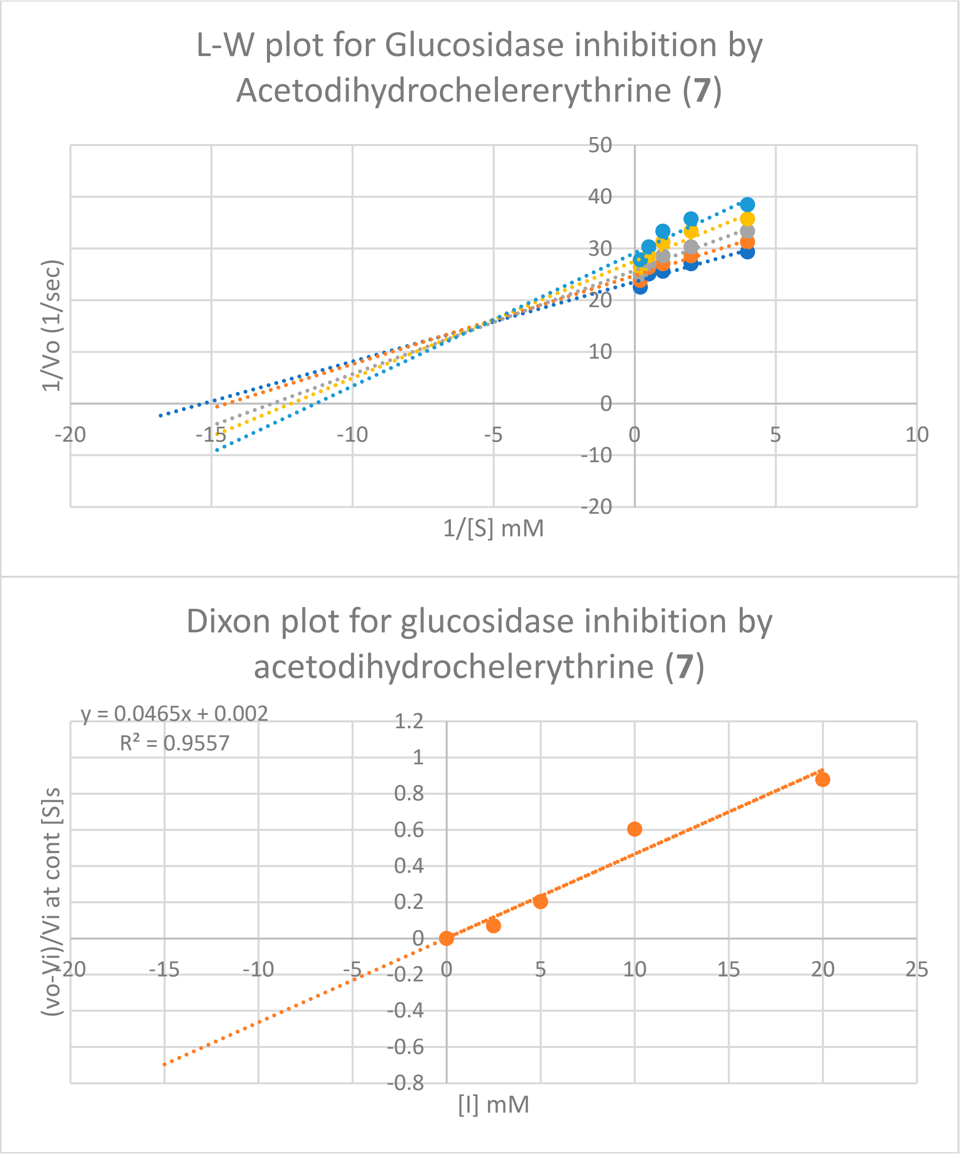

Figure S14: A- Double reciprocal (Lineweaver-Burk) plot for inhibition of α-glucosidase (mixed inhibition, Varied Km); B- Dixon plot for 6-Acetonyldihydrochelerythrine (**7**), Ki = **4.73 mM.**

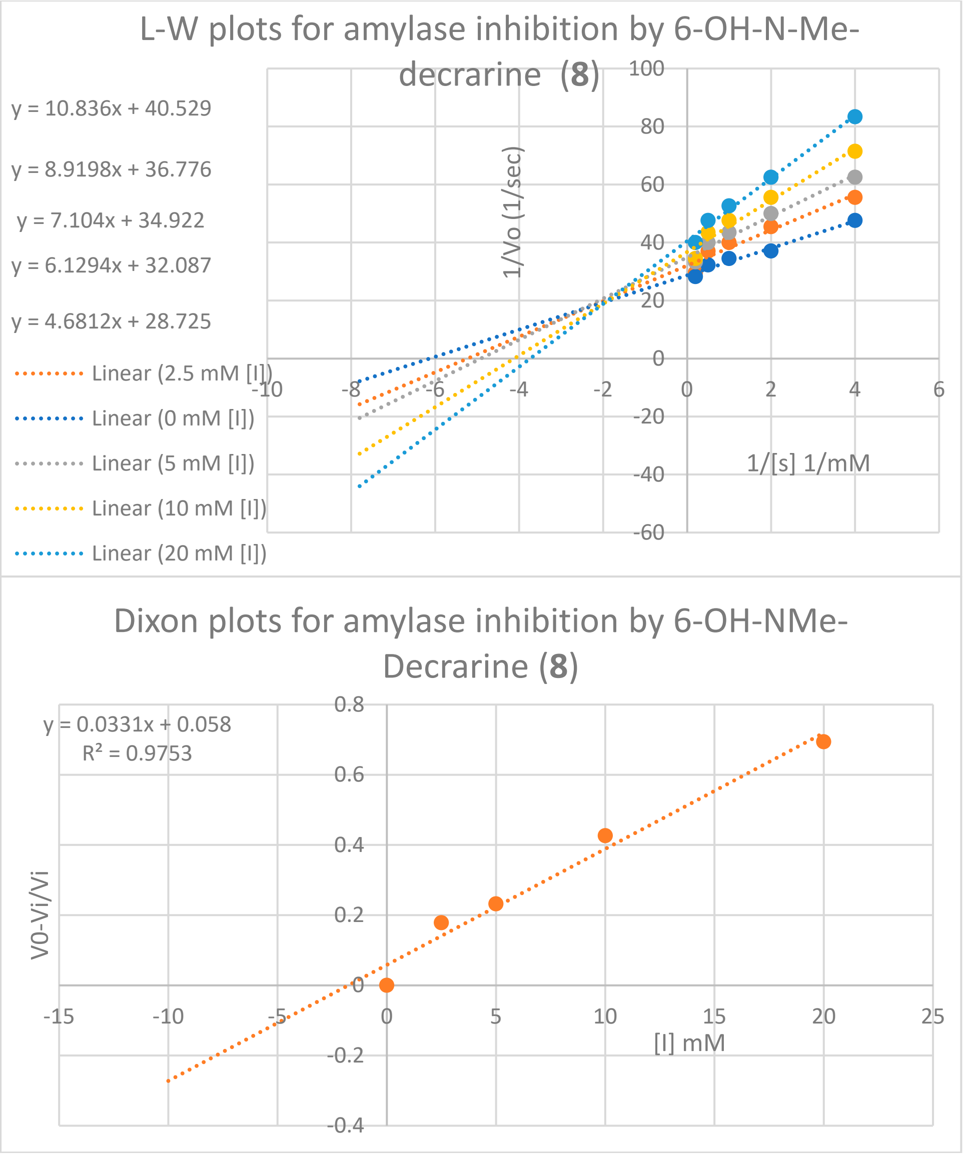

Figure S15: A- Double reciprocal (Lineweaver-Burk) plot for inhibition of α-amylase (mixed inhibition, varied Km); B- Dixon plot for 6-hydroxy-N-methyldecarine (**8**), Ki = **3.10 mM.**

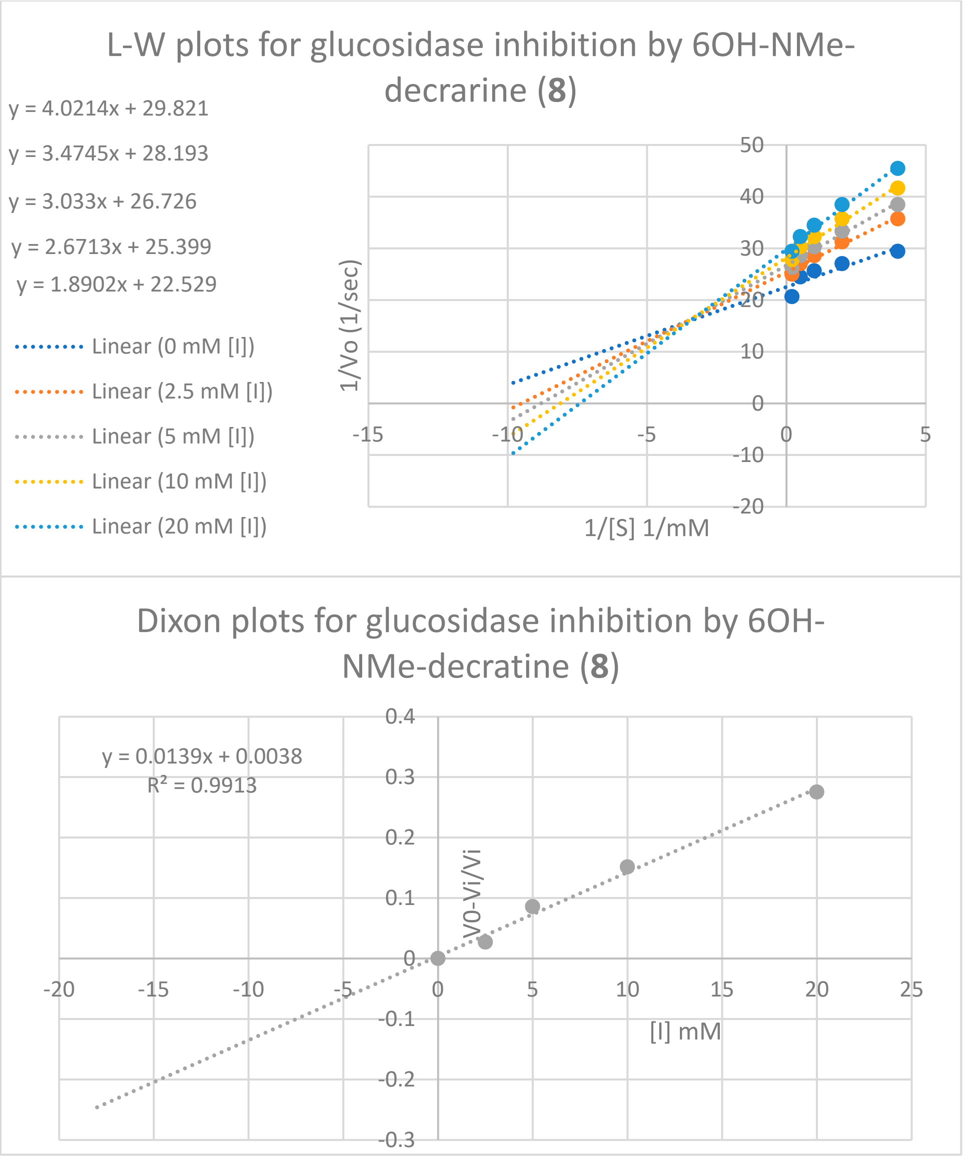

Figure S16: A- Double reciprocal (Lineweaver-Burk) plot for inhibition of α-glucosidase (mixed inhibition, varied Km); B- Dixon plot for 6-hydroxy-N-methyldecarine (**8**), Ki = **9.17 mM.**

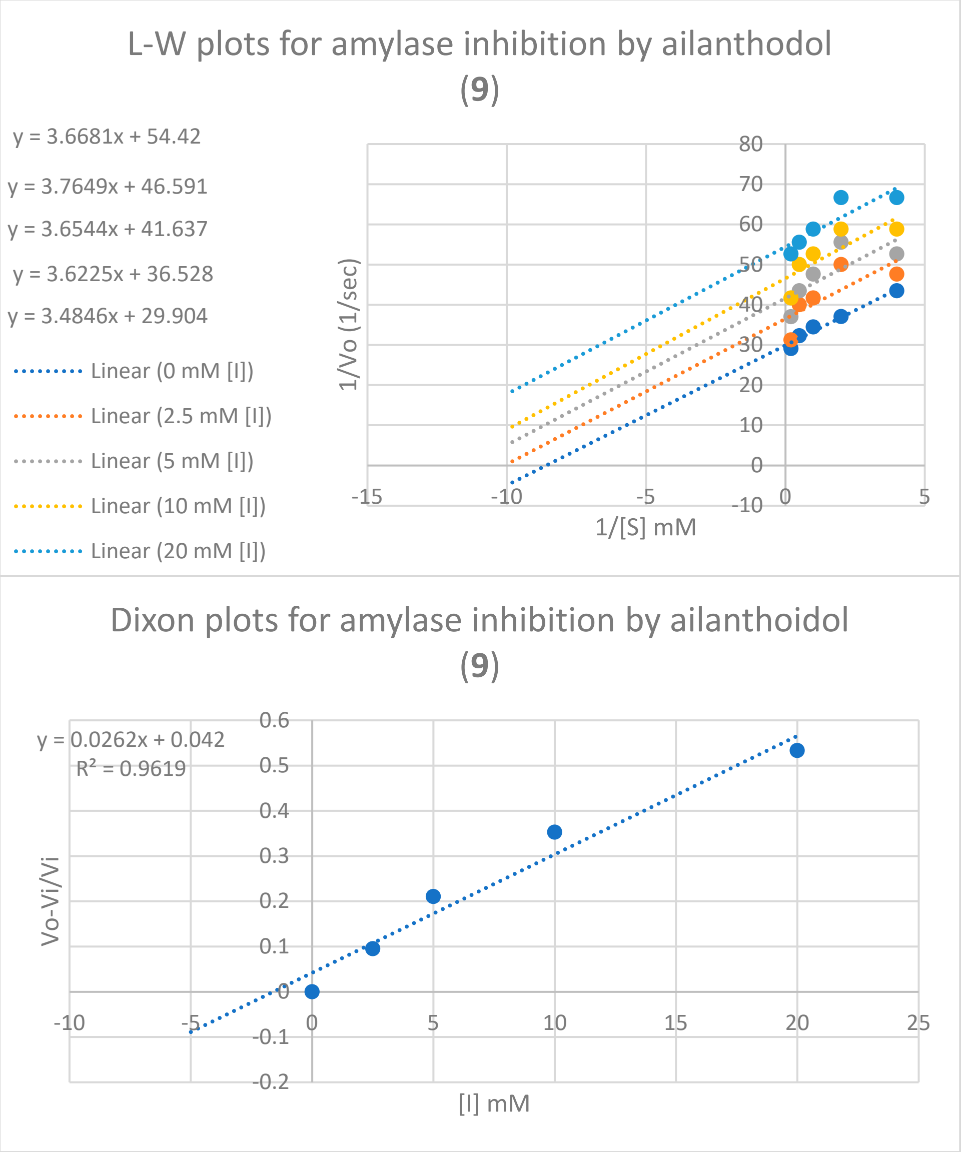

Figure S17: A- Double reciprocal (Lineweaver-Burk) plot for inhibition of α-amylase (un-competitive inhibition, varied Km); B- Dixon plot for Ailanthodial (**9**), Ki = **26.28 mM.**

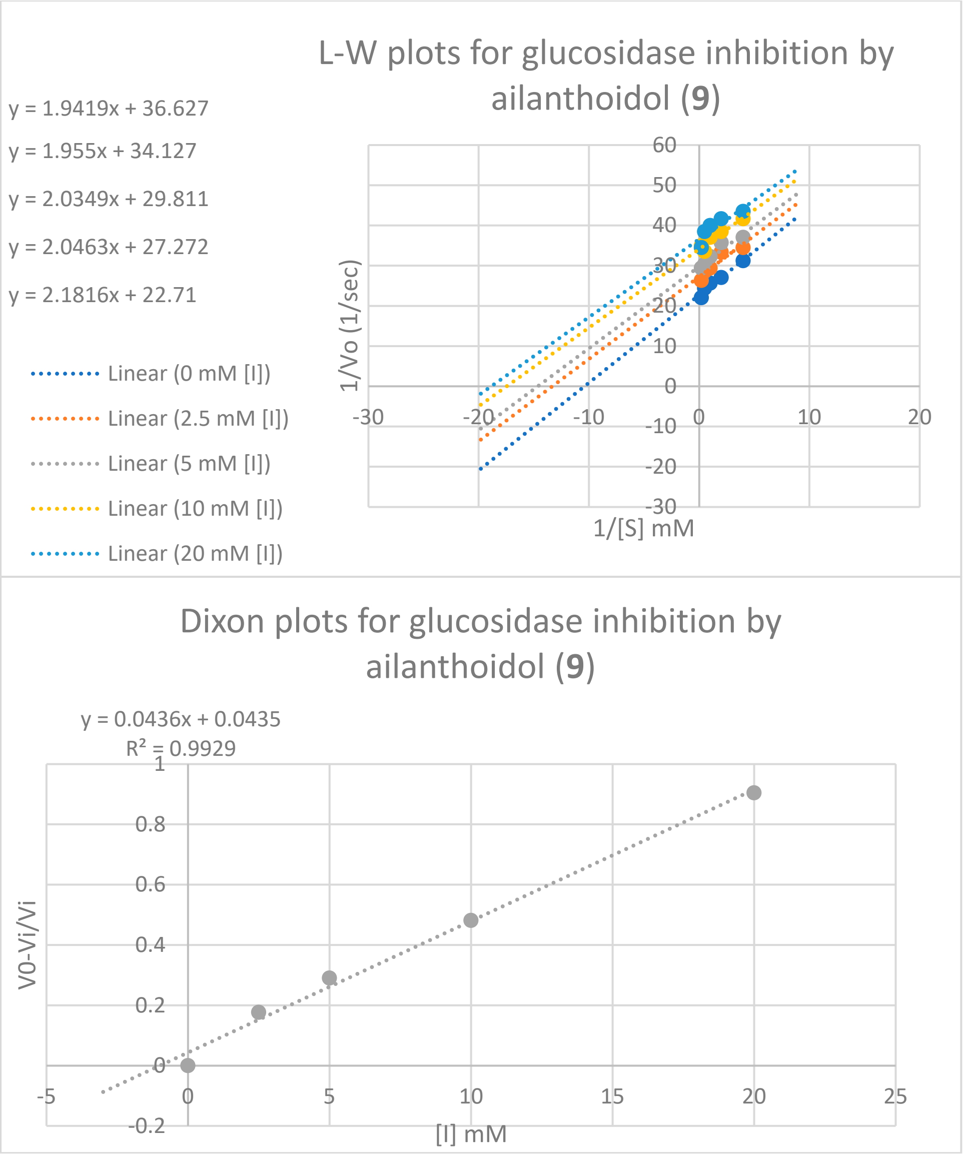

Figure S18: A- Double reciprocal (Lineweaver-Burk) plot for inhibition of α-glucosidase (non-competitive inhibition, Km = 5.53 mM); B- Dixon plot for Ailanthoidol (9), Ki = **20.63 mM.**

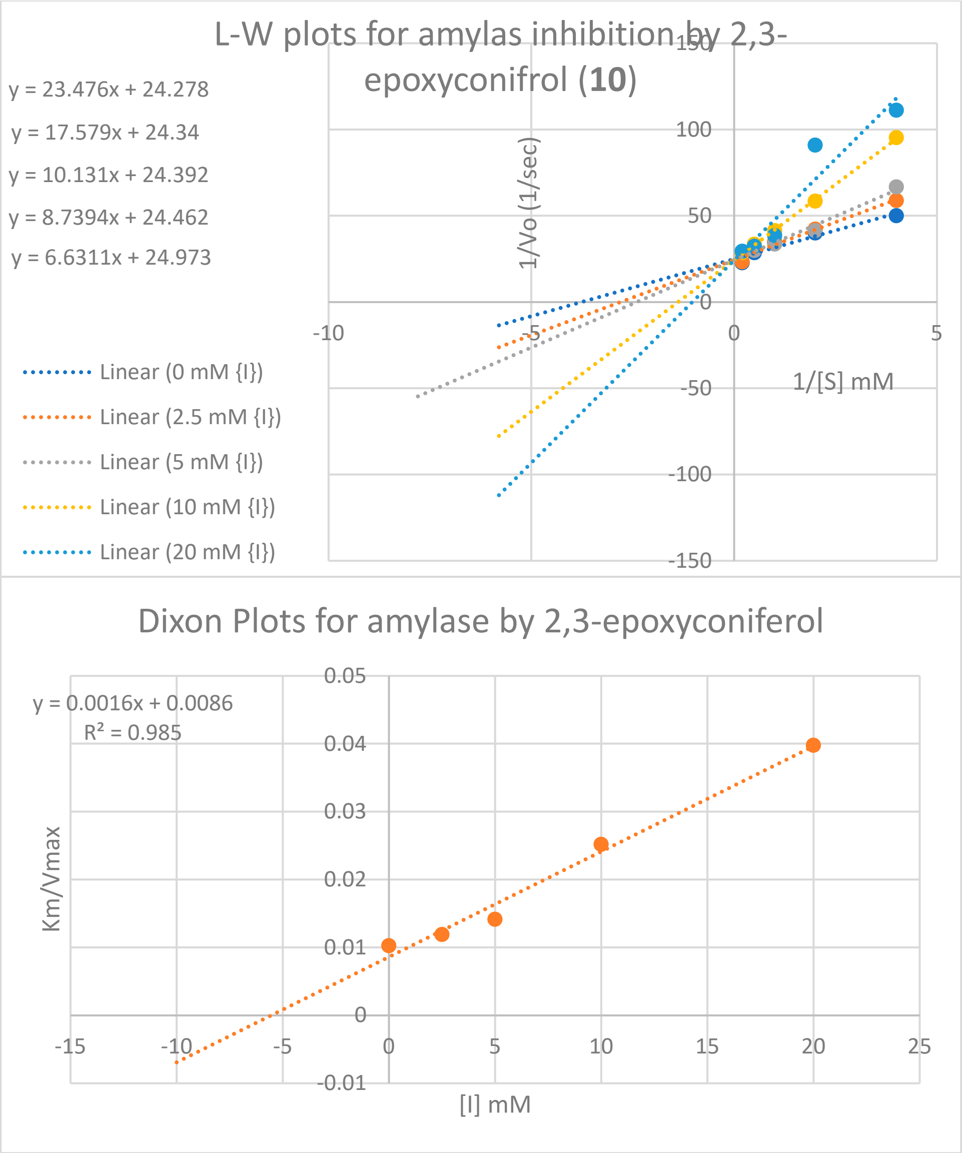

Figure S19: A- Double reciprocal (Lineweaver-Burk) plot for inhibition of α-amylase (competitive inhibition, $1/V_{max}$ = 24.47 sec^{-1}); B- Dixon plot for 2,3-epoxy-6,7-methylenedioxyconiferol (**10**), K_i = **5.54 mM.**

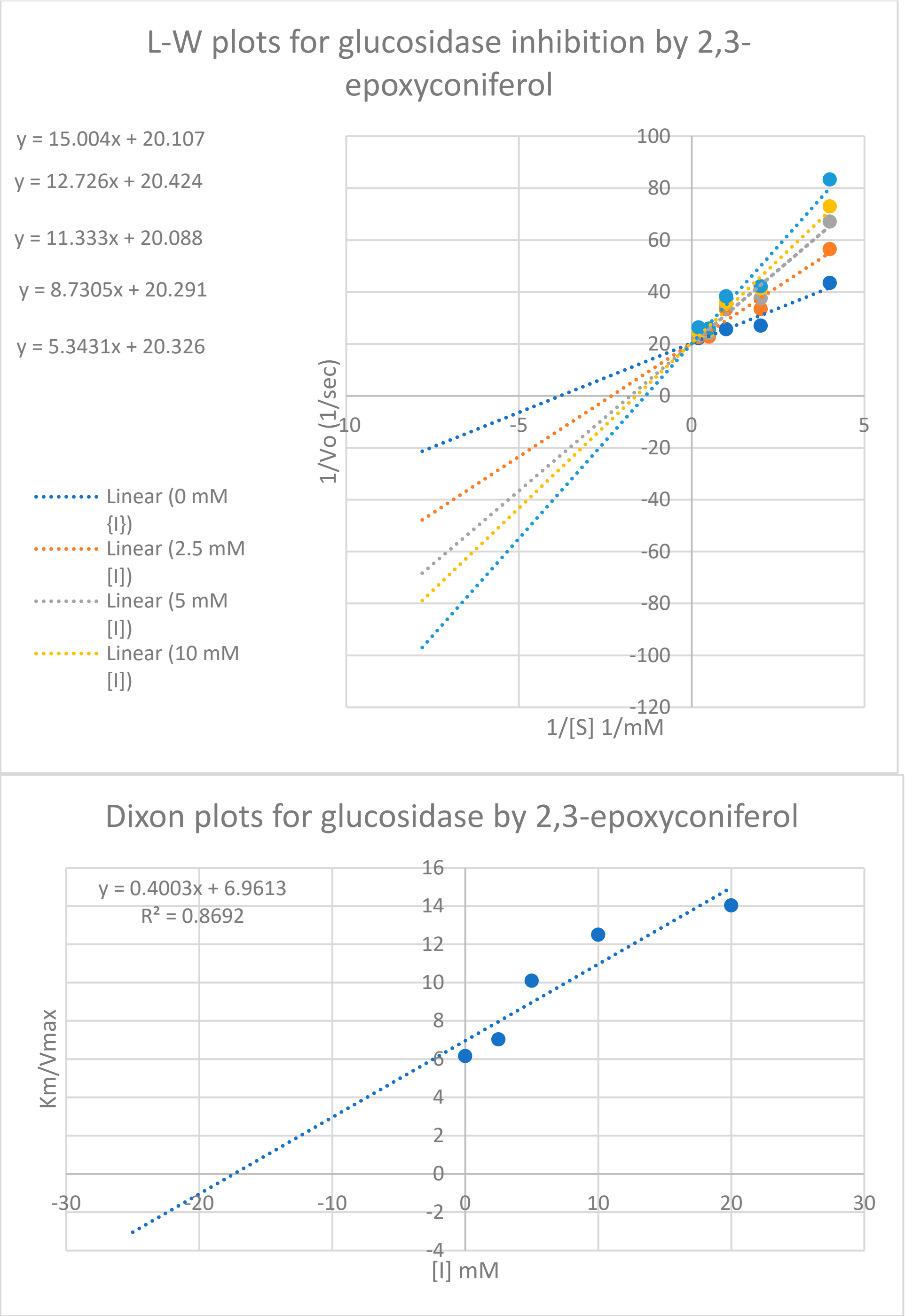

Figure S20: A- Double reciprocal (Lineweaver-Burk) plot for inhibition of α-glucosidase (competitive inhibition, $1/V_{max}$ = 20.25 sec^{-1}); B- Dixon plot for 2,3-epoxy-6,7-methylenedioxyconiferol (**10**), K_i = **17.21 mM.**

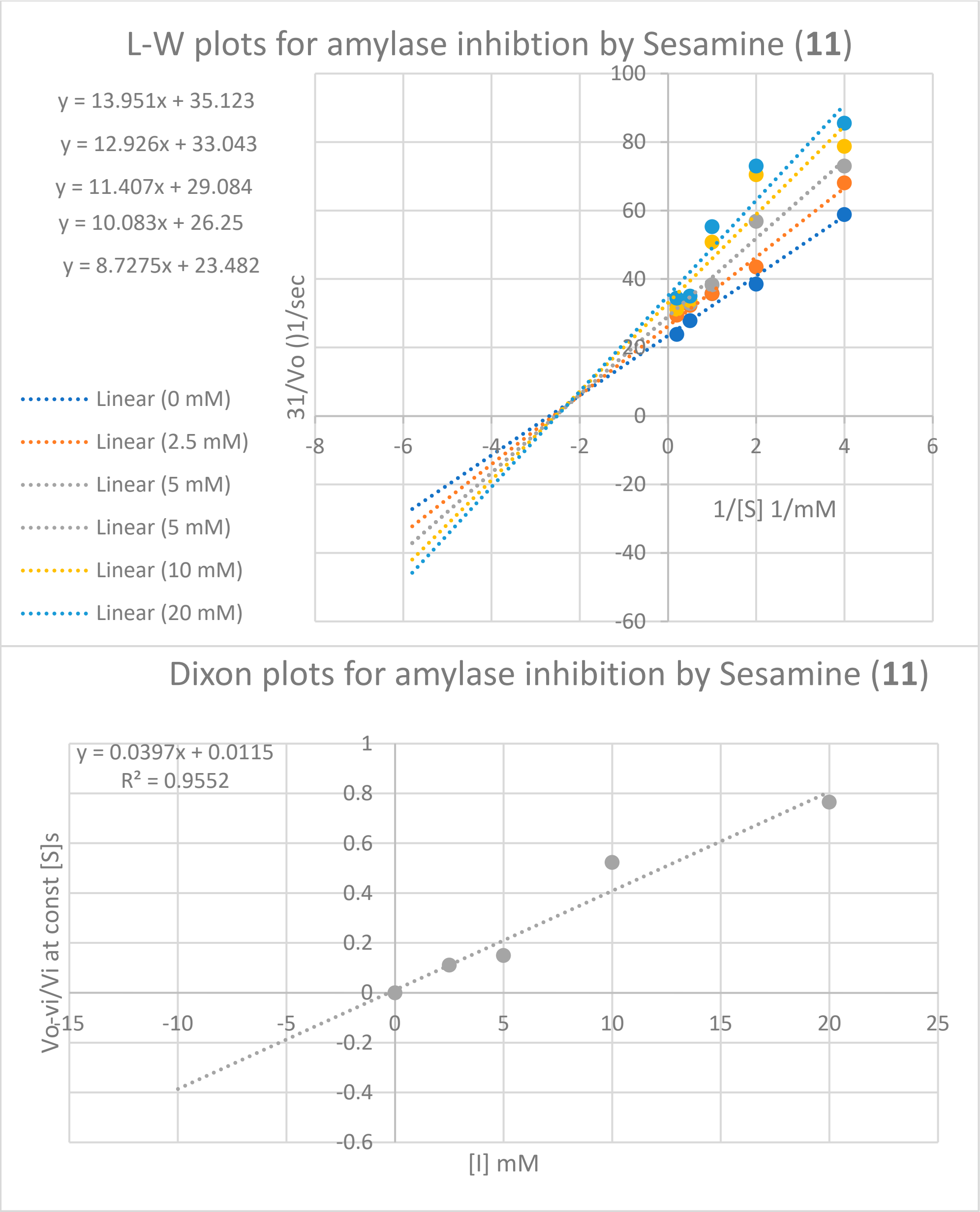

Figure S21: A- Double reciprocal (Lineweaver-Burk) plot for inhibition of α-amylase (non-competitive inhibition, Km = 2.69 mM); B- Dixon plot for Sesamine (**11**), Ki = **12.53 mM.**

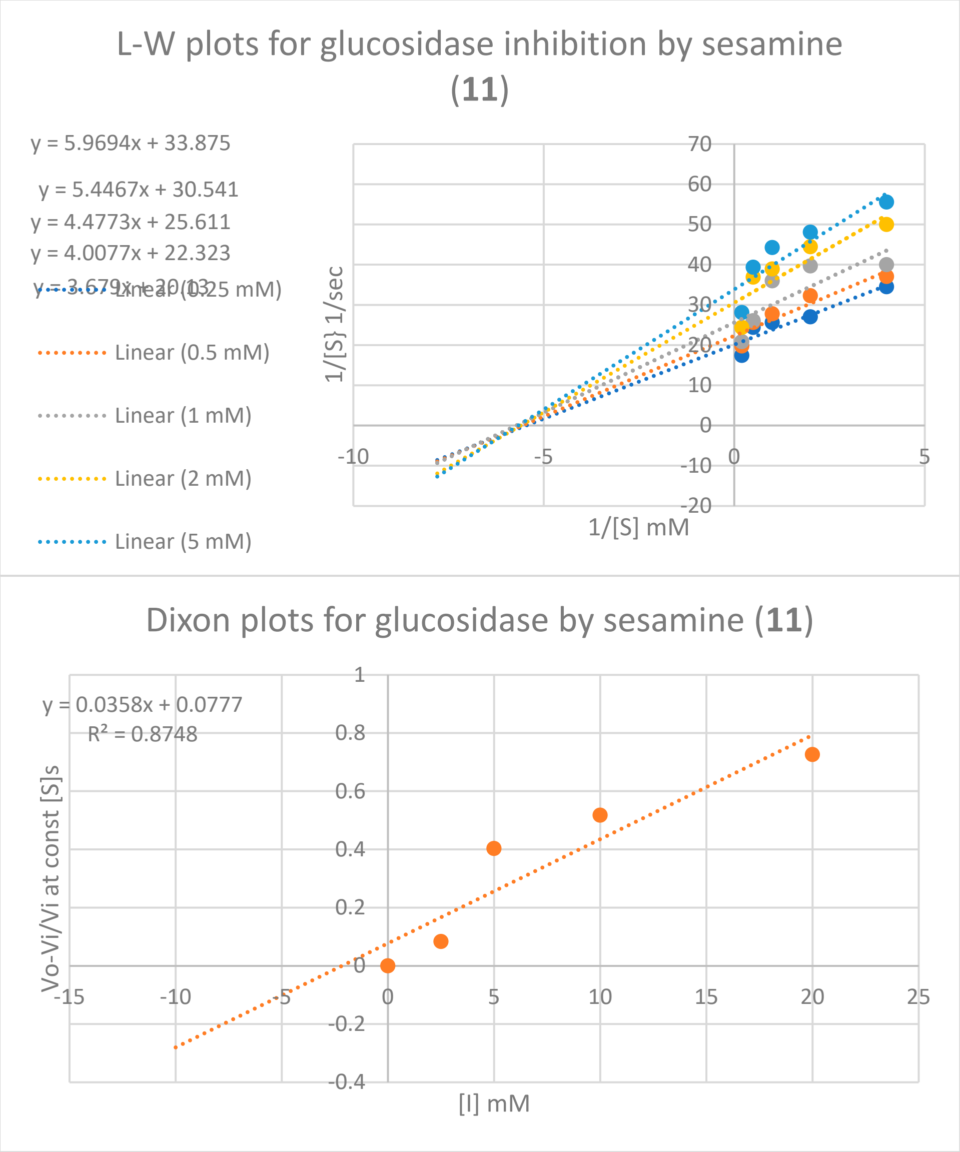

Figure S22: A- Double reciprocal (Lineweaver-Burk) plot for inhibition of *α*-glucosidase (non-competitive inhibition, Km = 5.61 mM); B- Dixon plot for Sesamine (**11**), Ki = **24.33 mM.**

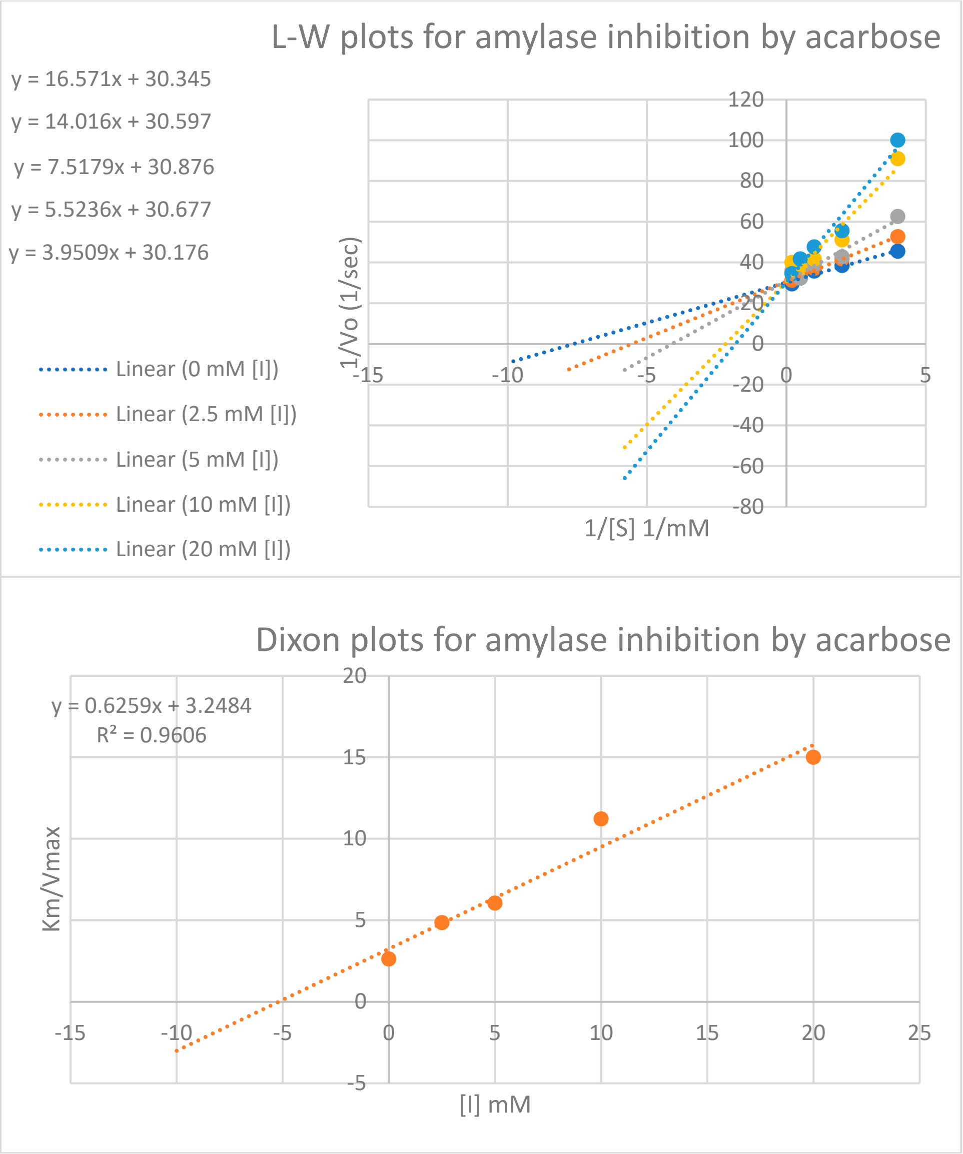

Figure S23: A- Double reciprocal (Lineweaver-Burk) plot for inhibition of α-amylase (competitive inhibition, $1/V_{max}$ = 30.45 sec^{-1}); B- Dixon plot for acarbose K_i = 6.14 **mM.**

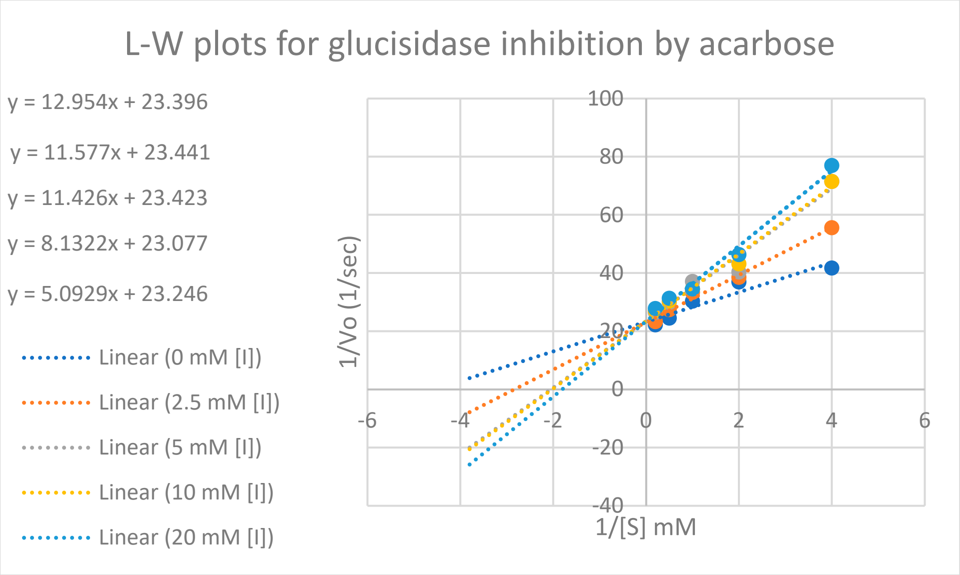

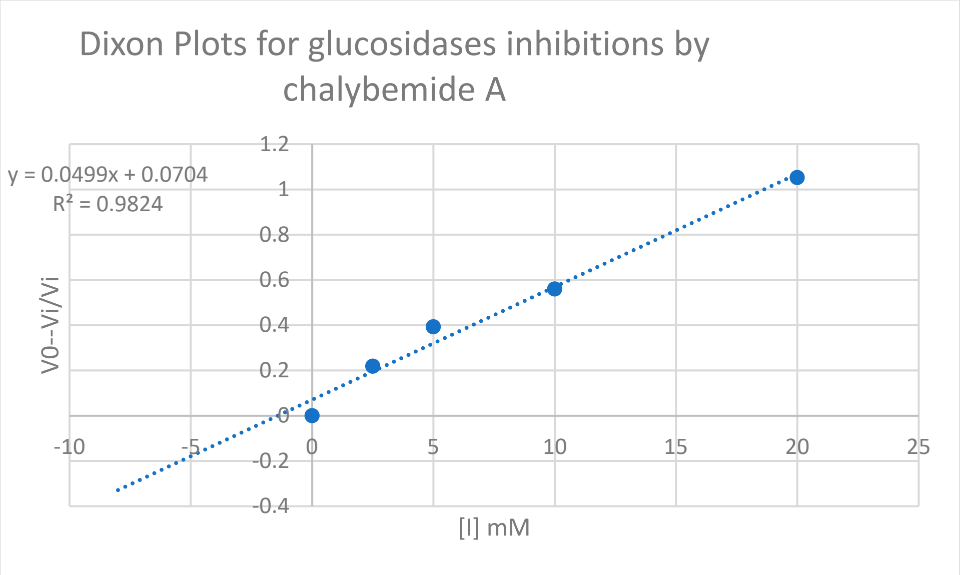

Figure S24: A- Double reciprocal (Lineweaver-Burk) plot for inhibition of α-glucosidase (competitive inhibition, $1/V_{max}$ = 23.32 sec^{-1}); B: Dixon plot for Acarbose, Ki = **22.40 mM.**

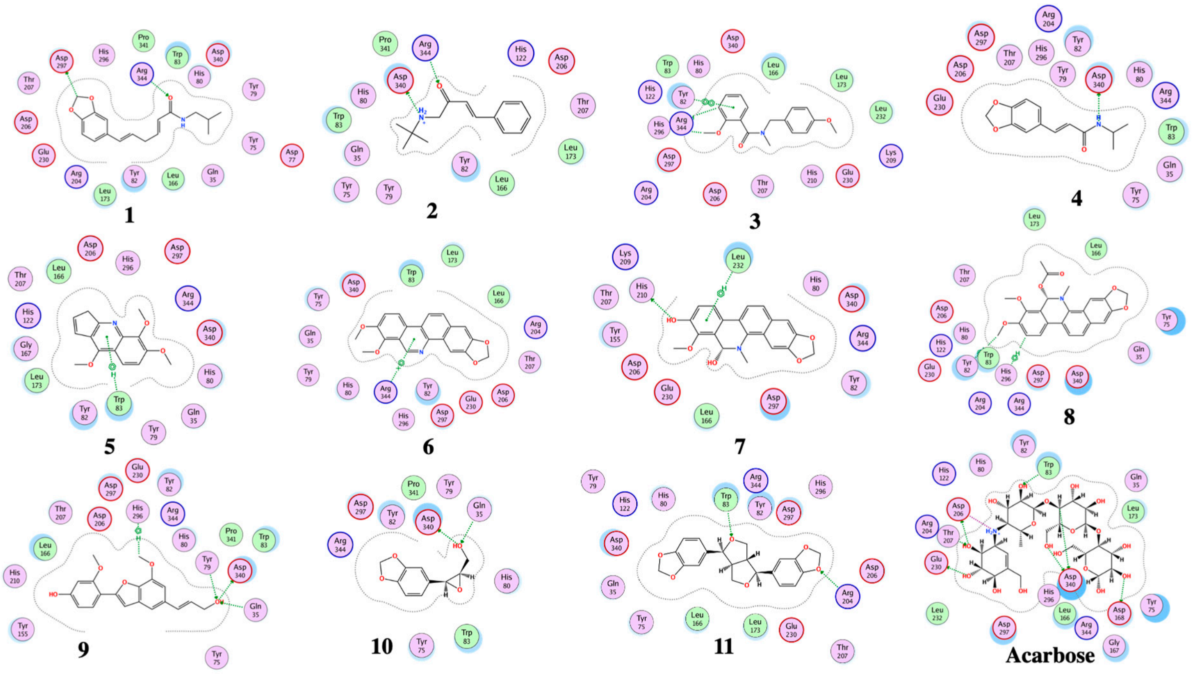

Figure S25: 2D interactions of compounds 1-11 and the reference Acarbose with α-amylase enzyme (PDB ID: 7TAA).

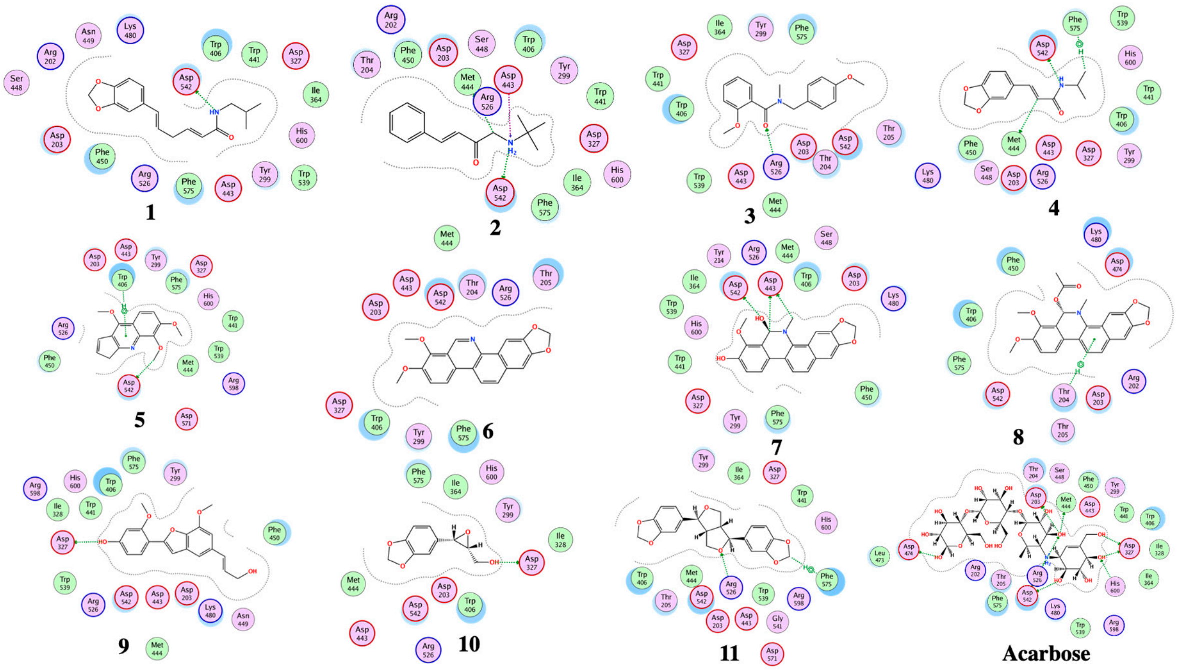

Figure S26: 2D interactions of compounds 1-11 and the reference Acarbose with α-glucosidase enzyme (PDB ID: 2QMJ).

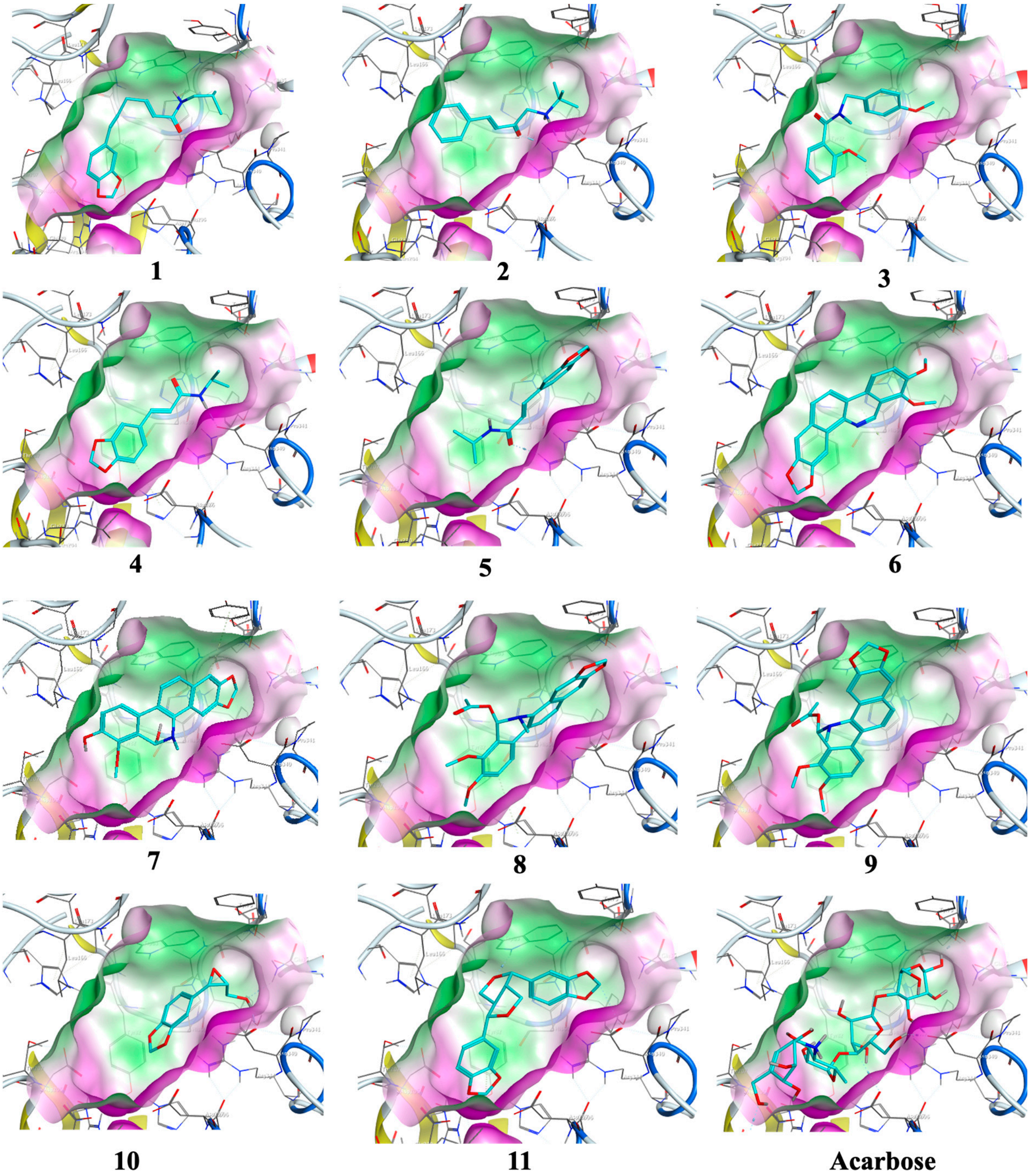

Figure S27: Docking conformation with the highest score for compounds **1-11** and the reference acarbose (carbon atoms in cyan) in the binding site of a-amylase (ID: 7TAA). Lipophilic areas on the molecular surface are colored green, while hydrophilic areas are colored purple.

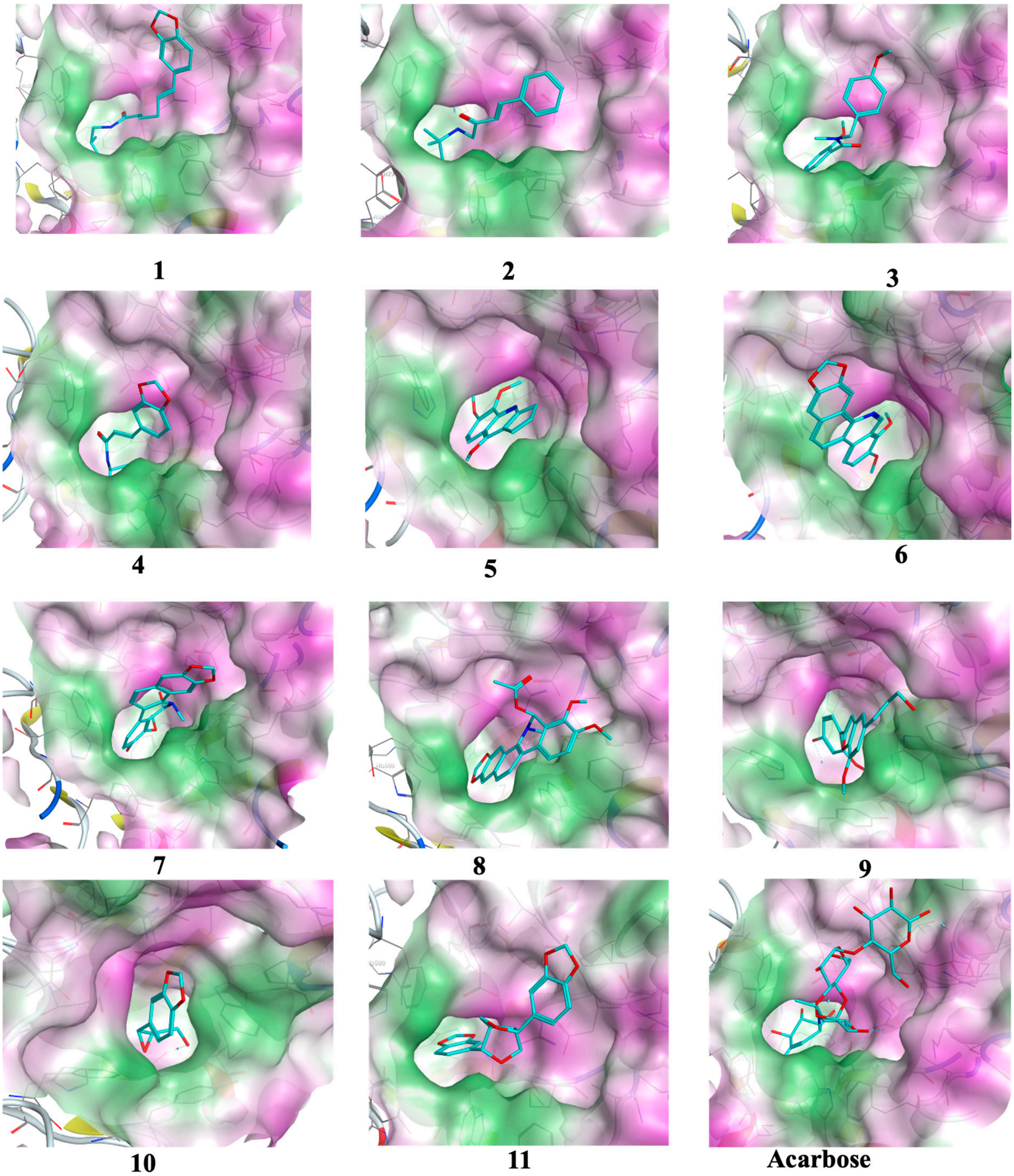

Figure S28: Docking conformation with the highest score for compounds 1-11 and the reference acarbose (carbon atoms in cyan) in the binding site of α-glucosidase (ID: 2QMJ. Lipophilic areas on the molecular surface are coloured green, while hydrophilic areas are coloured purple.
